# Mechanistic reconciliation of community and invasion ecology

**DOI:** 10.1002/ecs2.3359

**Published:** 2021-02-10

**Authors:** Guillaume Latombe, David M. Richardson, Melodie A. McGeoch, Res Altwegg, Jane A. Catford, Jonathan M. Chase, Franck Courchamp, Karen J. Esler, Jonathan M. Jeschke, Pietro Landi, John Measey, Guy F. Midgley, Henintsoa O. Minoarivelo, James G. Rodger, Cang Hui

**Affiliations:** ^1^ BioInvasions, Global Change Macroecology‐Group Department of Botany and Biodiversity Research University Vienna Rennweg 14 Vienna 1030 Austria; ^2^ Centre for Invasion Biology Department of Botany and Zoology Stellenbosch University Stellenbosch 7600 South Africa; ^3^ School of Biological Sciences Monash University Clayton Victoria 3800 Australia; ^4^ Statistics in Ecology, Environment and Conservation Department of Statistical Sciences University of Cape Town Rondebosch 7701 South Africa; ^5^ Department of Geography King’s College London WC2B 4BG London UK; ^6^ German Centre for Integrative Biodiversity Research (iDiv) Halle‐Jena‐Leipzig Deutscherplatz 5e Leipzig Germany; ^7^ Department of Computer Sciences Martin Luther University Halle (Saale) Germany; ^8^ Université Paris‐Saclay Ecologie Systématique et Evolution CNRS AgroParisTech Orsay 91405 France; ^9^ Department of Conservation Ecology & Entomology and Centre for Invasion Biology Stellenbosch University Private Bag x1 Matieland 7602 South Africa; ^10^ Leibniz Institute of Freshwater Ecology and Inland Fisheries (IGB) Müggelseedamm 310 Berlin 12587 Germany; ^11^ Freie Universität Berlin Department of Biology, Chemistry, Pharmacy Institute of Biology Königin‐Luise‐Str. 1‐3 Berlin 14195 Germany; ^12^ Berlin‐Brandenburg Institute of Advanced Biodiversity Research (BBIB) Königin‐Luise‐Str. 2‐4 Berlin 14195 Germany; ^13^ Centre for Invasion Biology Department of Mathematical Sciences Stellenbosch University Stellenbosch 7600 South Africa; ^14^ Global Change Biology Group Department of Botany and Zoology Stellenbosch University Stellenbosch 7600 South Africa; ^15^ Biodiversity Informatics Unit African Institute for Mathematical Sciences Cape Town 7945 South Africa

**Keywords:** community ecology, hypothesis, invasion ecology, model, process, theory

## Abstract

Community and invasion ecology have mostly grown independently. There is substantial overlap in the processes captured by different models in the two fields, and various frameworks have been developed to reduce this redundancy and synthesize information content. Despite broad recognition that community and invasion ecology are interconnected, a process‐based framework synthesizing models across these two fields is lacking. Here we review 65 representative community and invasion models and propose a common framework articulated around six processes (dispersal, drift, abiotic interactions, within‐guild interactions, cross‐guild interactions, and genetic changes). The framework is designed to synthesize the content of the two fields, provide a general perspective on their development, and enable their comparison. The application of this framework and of a novel method based on network theory reveals some lack of coherence between the two fields, despite some historical similarities. Community ecology models are characterized by combinations of multiple processes, likely reflecting the search for an overarching theory to explain community assembly and structure, drawing predominantly on interaction processes, but also accounting largely for the other processes. In contrast, most models in invasion ecology invoke fewer processes and focus more on interactions between introduced species and their novel biotic and abiotic environment. The historical dominance of interaction processes and their independent developments in the two fields is also reflected in the lower level of coherence for models involving interactions, compared to models involving dispersal, drift, and genetic changes. It appears that community ecology, with a longer history than invasion ecology, has transitioned from the search for single explanations for patterns observed in nature to investigate how processes may interact mechanistically, thereby generating and testing hypotheses. Our framework paves the way for a similar transition in invasion ecology, to better capture the dynamics of multiple alien species introduced in complex communities. Reciprocally, applying insights from invasion to community ecology will help us understand and predict the future of ecological communities in the Anthropocene, in which human activities are weakening species’ natural boundaries. Ultimately, the successful integration of the two fields could advance a predictive ecology that is urgently required in a rapidly changing world.

## Introduction

The fields of community and invasion ecology have traditionally had different, but interrelated scopes. Community ecology aims primarily to explain how multiple species can coexist. Its scope encompasses the origin, evolution, maintenance, and dynamics of biodiversity within communities in diverse environments (Vellend 2016, Leibold and Chase 2017). Invasion ecology, on the other hand, focuses on species introduced to novel environments by humans (termed alien species) and asks questions relating to how populations of alien species spread and interact with other species in these environments. Invasion ecology has a strong applied focus and has grown largely from concepts in population ecology; most early studies of invasions focused on understanding and controlling particular invasive species with major impacts. Except for studies of enemies or mutualists of alien species, invasion ecology has largely progressed independently from community ecology, at least until the last decade or two (Hui and Richardson 2017).

Despite their largely separate historical trajectories, it is now accepted that community and invasion ecology are not independent from each other: Once a species is introduced to a novel environment, it interacts with the local community and forms part of the network of interacting species (Hui and Richardson 2019*a*). In addition, communities are often invaded by multiple alien species which, once established, can become impossible to control and, in some cases, become permanent members of the landscape, creating novel ecosystems (Hobbs et al. 2014). The traditional perspective of single alien species interacting only with specific native species, or with the abiotic environment, clearly does not capture the complexity of multiple alien species interacting with each other, with multiple native species, and with abiotic factors in a spatially heterogeneous environment. Consequently, community ecology has repeatedly been proposed as a crucial framework for invasion ecology (Shea and Chesson 2002, MacDougall et al. 2009, Pearson et al. 2018). Correlative studies and meta‐analyses bridging both perspectives (e.g., Gaertner et al. 2009, Gallien and Carboni 2017) have shown that invasion ecology can benefit from insights that have accrued in community ecology regarding the coexistence of multiple species competing for limited resources and space, and the effects of disturbance and stochasticity on species persistence and coexistence. We will show here how merging insights from the two fields, through a mechanistic framework, creates a much‐needed integrative perspective in ecology, which will ultimately allow us to achieve accrued predictive power about the success or failure of biological invasions, but also to forecast changes in the structure of communities invaded by multiple alien species.

Reciprocally, biological invasions can be seen as a kind of perturbation to native communities. Invasions have been framed as biogeographical assays, providing unique opportunities to uncover the mechanisms that structure communities (Cadotte et al. 2006, Rouget et al. 2015). Biological invasions have also been shown to trigger regime shifts, altering multiple facets of ecological communities such that their new structures are hard, or impossible, to reverse (Gaertner et al. 2014). Biological invasions therefore have the potential to revolutionize our view of ecological communities and meta‐communities, from a closed system with coexisting species to an open system with a high rate of multi‐species propagule exchange through permeable boundaries and co‐evolving components (Frost et al. 2019, King and Howeth 2019, McGrannachan and McGeoch 2019, Hui and Richardson 2019*a*, *b*). Applying insights from invasion biology to community ecology will help us better understand and predict the future of ecological communities in the Anthropocene, in which human activities are weakening species’ natural boundaries.

Despite the clear interplay between the two fields, community and invasion ecology have developed their own sets of models, theories, and hypotheses. Community ecology tends to seek an overarching and universal theory of the assembly and maintenance of biodiversity, and heated debates arise when different models appear to contradict each other. This is exemplified by arguments around Hubbell’s (2001) Unified Neutral Theory of Biodiversity, which contradicts the well‐established niche theory (see Clark 2012 and Rosindell et al. 2012 for contrasting perspectives). The effect of spatial scale on community patterns further complicates the study of ecological communities (Chase et al. 2018), as does the fact that local ecological communities interact with each other within meta‐communities via propagule exchange between locations with different environmental conditions (Leibold and Chase 2017). Ecological communities are therefore complex and involve dynamic interactions among many organisms, each with their own traits and functions for the maintenance of biodiversity. To reduce complexity and redundancies in community ecology, Vellend (2016) proposed a conceptual framework based on four high‐level processes (dispersal, selection, speciation, and drift) that, he argued, described the fundamental dimensions of community ecology, thereby bringing coherence to the field.

Rather than searching for overarching models, most work in invasion ecology seeks to explain or predict how species perform in a recipient ecosystem outside of their native ranges and the impacts of such biological incursions (see also Catford et al. 2009, Jeschke and Heger 2018). Many of the models and hypotheses that have emerged in recent decades are nonetheless interrelated, and understanding how they relate to each other is not straightforward (Enders et al. 2018). Frameworks have therefore been proposed to structure the models and hypotheses of invasion biology, thereby contributing to the development of overarching theories. Catford et al. (2009), in particular, proposed classifying invasion models and hypotheses according to the combination of three key components: propagule pressure, the abiotic characteristics of the receiving ecosystem, and the biotic characteristics of the recipient community and of the alien species. Although emerging independently, this process‐based classification of invasion models and hypotheses maps onto the concept of dispersal, environmental, and biotic filters used to explain community assembly (Stokes and Archer 2010) and shares many similarities with Vellend’s (2016) conceptual framework of high‐level processes for community ecology.

A mechanistic (process‐based) framework unifying community and invasion ecology is yet to emerge. This is highlighted by the lack of a general model to predict spread and impacts of alien species and the response of recipient communities (Courchamp et al. 2017). Here, we collate and extend process‐based conceptual frameworks from both community and invasion ecology to better capture their interplay (Catford et al. 2009, Vellend 2016). We propose a set of processes that can be applied across community (including metacommunity) and invasion models (Tables 1, 2), which we use to examine, characterize, compare, and synthesize a representative set of existing models at local and regional scales (given the lack of consensus in ecology about what qualifies as a theory, see Marquet et al. 2014, or even a hypothesis, see Murray 2004, we will use “community model” and “invasion model” as overarching terms for simplicity and coherence through the article). Based on the resulting process characterization, we also match community and invasion models and analyze the results using a novel method based on network theory, to complete the conceptual picture of the two fields and identify alignments and gaps. We see this as a crucial first step toward a synthesis enabling both fields to maximize benefits from one another, therefore providing novel perspectives to improve the ability to address interrelated issues in community and invasion ecology in the current context of global changes, and to move toward predictive models supporting robust management actions for nature conservation and invasion control in a holistic fashion.

**Table 1:** Community models and their classification as process‐ or pattern‐based (expanding on Vellend 2016).

ID	Name	Description	Reference(s)	Classification
C1	Adaptive dynamics (AD)	Mutation limited evolution of phenotypic traits driven by ecological interactions determines the structure of a community.	Fussmann et al. (2007)	Process
C2	Bottom‐up regulation (BUR)	Community composition is driven by resources (lower trophic levels).	Oksanen et al. 1981, Matson and Hunter (1992)	Process
C3	Colonization‐competition trade‐off / patch dynamics (CCT/PD)	Good colonizers (dispersers) are bad competitors and reciprocally.	Levins and Culver (1971)	Process
C4	Community Assembly Phase Space (CAPS)	The combination of neutral and niche processes can generate structures that lie outside of the neutral‐niche continuum due to feedbacks.	Latombe et al. (2015)	Process
C5	Competitive exclusion principle (CE)	Two species competing for the exact same resource cannot coexist because one will inevitably have a slight advantage.	Gause (1934)	Process
C6	Ecosystem engineering (EE)	Community structure is influenced by severe effects of one species on the abiotic environment.	Jones et al. (1994)	Process
C7	Enemy‐mediated coexistence (EMC)	Enemies (predators, pathogens, etc.) have a larger effect on the most abundant species; that is, negative density dependence.	Holt et al. (1994)	Process
C8	Equalizing/stabilizing criteria (ESC)	Coexistence between species is permitted by (i) a reduction in fitness difference and (ii) niche differentiation between species.	Chesson (2000a)	Process
C9	Facilitation‐based theory (FBT)	Community structure is explained by positive interactions between species, which promotes coexistence.	Bruno et al. (2003)	Process
C10	Genetic feedback (GF)	Natural selection enables a species with poor interaction ability to change its interaction mechanism and to recover.	Pimentel (1968)	Process
C11	Hump‐shaped diversity‐productivity hypothesis (HSDPH)	Low and high productivity generate stress and competitive exclusion, which reduces diversity, while constraints are relaxed at intermediate productivity.	Grime (1973)	Process
C12	Intermediate disturbance hypothesis (IDH)	Intermediate disturbance decrease competition and therefore the dominance of strong competitors.	Grime (1973), Connell (1978)	Process
C13	Intransitive competition (IC)	Each species is competitively superior to some and inferior to others, similar to rock‐paper‐scissors.	Gilpin (1975)	Process
C14	Janzen‐Connell effects (JC)	Species‐specific enemies accumulate around adult trees, preventing local regeneration of that species.	Connell (1970), Janzen (1970)	Process
C15	Mass effect (ME)	Colonization from occupied sites enables a species to survive in a site with unfavorable environment.	Holyoak et al. (2005), Leibold and Chase (2017)	Process
*C16*	*Maximum Entropy Theory of Ecology (METE)*	*Community patterns are generated by maximizing information entropy under constraints on area (A), species richness (S), species abundance (N), and total metabolic rate of the individuals (E)–ASNE model*.	*Harte (* *2011* *)*	*Statistical property*
*C17*	*Multiple stable equilibria (MSE)*	*Positive feedbacks and perturbation/stochasticity can lead the community to switch between different equilibria*.	Scheffer (2009)	*Pattern (Process)*
C18	Neutral theory (NeT)	All species are equivalent from a per capita perspective and species coexistence emerges from immigration and speciation.	Hubbell (2001)	Process
C19	Neutral‐niche continuum (NNC)	Communities have structures that lie between the structures generated by pure neutral (no interactions) and pure niche (only interactions) processes.	Gravel et al. (2006)	Process
C20	Niche theory (NiT)	Umbrella term for models based on interaction processes, biotic or abiotic.	Chase and Leibold (2003)	Process
*C21*	*Priority effect (PE)*	*Initial colonists of a given site inhibit or facilitate the establishment of other species, for different possible reasons*.	*Fukami (* *2010* , *2015* *)*	*Pattern*
C22	R* theory (R*)	When dealing with multiple resources, species with the lowest R* (lowest level of resources at which it can persist) outcompete other species.	Tilman (1982)	Process
C23	Relative nonlinearity of competition (RNC)	Interactions with resources fluctuates temporally due to the impact on resource levels by the species, resulting in non‐linear fitness responses to resource levels.	Armstrong and McGehee (1980)	Process
C24	Spatial storage effect (SSE)	Species have different niches and can persist where the environment is not optimal (e.g., through seed banks). In addition, per capita intraspecific competition is greatest at high abundance, and interspecific competition is greatest at low abundance.	Chesson (2000b)	Process
C25	Species pool hypothesis (SPH)	Local community diversity is limited by the regional species pool, which is determined by regional and historical interactions, dispersal, speciation, and drift processes.	Taylor et al. (1990)	Process
C26	Species sorting (SS)	Species differ in their fitness in different abiotic environments (similar to niche theory but abiotic only).	Holyoak et al. (2005), Leibold and Chase (2017)	Process
C27	Species‐energy theory (SET)	Species richness is driven by a trade‐off between immigration from a global species pool and local extinction, which is driven by available energy (similar to TIB with energy instead of area).	Wright (1983)	Process
C28	Stochastic niche theory (SN)	Niche theory incorporating drift and propagule pressure.	Tilman (2004)	Process
C29	Succession theory (ST)	Umbrella term for community dynamics, for example, after disturbance, incorporating all processes but speciation.	Pickett et al. (1987)	Process
C30	Temporal storage effect (TS)	Species have different niches and can persist when the environment is not optimal (e.g., through seed banks). In addition, per capita intraspecific competition is greatest at high abundance, and interspecific competition is greatest at low abundance.	Chesson (2000b)	Process
C31	Theory of island biogeography (TIB)	Species richness is driven by a trade‐off between immigration from a global species pool and local extinction, which is driven by area.	MacArthur and Wilson (1967)	Process
C32	Top‐down regulation (TDR)	Community composition is driven by predators (higher trophic levels).	Matson and Hunter (1992)	Process

Notes

Italics denotes models that are not process based under the strict characterization. The multiple stable equilibria models are considered to be pattern‐based under a strict characterization scheme, and process‐based under the inclusive characterization only, as indicated between parenthesis.

**Table 2:** Invasion models and their classification as process‐ or pattern‐based (adapted from Catford et al. 2009 and Enders et al. 2018).

ID	Name	Description	Reference(s)	Classification
I1	Adaptation (A)	The invasion success of alien species depends on their pre‐introduction adaptation to the conditions in the exotic range. Alien species that are related to native species are more successful in this adaptation.	Duncan and Williams (2002)	Process
*I2*	*Biotic acceptance aka “the rich get richer” (BA)*	*Ecosystems with more native species are more invaded. This can be due to multiple processes*.	*Stohlgren et al. (* *1998* *)*	*Pattern (Process)*
I3	Biotic indirect effects (BID)	Combinations of cross‐guild and potentially abiotic processes can lead to indirect biotic interactions between species of the same guild.	Callaway et al. (2004)	Process
*I4*	*Biotic resistance aka diversity‐invasibility hypothesis (BR)*	*Ecosystems with high richness get less invaded than ecosystems with lower richness. This can be due to multiple processes*.	*Elton (* *1958* *)*, *Levine and D’Antonio (* *1999* *)*	*Pattern (Process)*
I5	Darwin’s naturalization (DN)	The invasion success of alien species is higher in areas with few phylogenetically close species than in areas with many phylogenetically close species.	Darwin (1859)	Process
*I6*	*Disturbance (DS)*	*The invasion success of alien species is higher in highly disturbed than in relatively undisturbed ecosystems*.	*Elton (* *1958* *)*, *Hobbs and Huenneke (* *1992* *)*	*Pattern (Process)*
I7	Dynamic equilibrium (DEM)	The establishment of an alien species depends on natural fluctuations of the ecosystem, which influences the competition of local species.	Huston (1979)	Process
I8	Empty niche (EN)	The presence of empty niches increases the likelihood of alien species with adequate niches to invade.	MacArthur (1970)	Process
I9	Enemy inversion (EI)	Introduced enemies of alien species are less harmful for them in the exotic than the native range, due to altered biotic and abiotic conditions.	Colautti et al. (2004)	Process
I10	Enemy of my enemy (EE)	Introduced enemies of an alien species are more harmful to the native than to the alien species, giving the alien species a competitive advantage.	Eppinga et al. (2006)	Process
I11	Enemy reduction (ERD)	Enemies are less frequent in the introduced range, resulting in being less harmful. Similar to enemy inversion but due to population abundance than to actual predation mechanism.	Colautti et al. (2004)	Process
I12	Enemy release (ER)	Enemies are absent in the introduced range, resulting in fitness improvement for the alien species.	Keane and Crawley (2002)	Process
I13	Environmental heterogeneity (EVH)	A highly heterogeneous environment provides more niche therefore more invasion opportunities (similar to the empty niche for the abiotic environment).	Melbourne et al. (2007)	Process
I14	Evolution of increased competitive ability (EICA)	Release from natural enemies leads alien species to allocate more energy in growth and/or reproduction (this re‐allocation is due to genetic changes), resulting in a competitive advantage.	Blossey and Notzold (1995)	Process
I15	Global competition (GC)–equivalent to Sampling (SP)	A large number of different alien species is more successful than a small number because there is more chance than at least one of them will outcompete native species due to interaction processes.	Crawley et al. (1999), Alpert (2006)	Process
I16	Habitat filtering (HF)	The invasion success of alien species whose niche fits the abiotic environment in the introduced area is high.	Darwin (1859), Melbourne et al. (2007)	Process
*I17*	*Human commensalism (HC)*	*Species living in close proximity to humans are more successful in invading new areas than other species*.	Jeschke and Strayer (2006)	*Pattern*
*I18*	*Ideal weed (IW)*	*The invasion success of an alien species is determined by its specific traits, such as life‐history traits*.	*Baker (* *1965* *)*, *Rejmánek and Richardson (* *1996* *)*	*Trait‐based*
I19	Increased resource availability (IRA)	High resource availability increases the invasion success of alien species.	Sher and Hyatt (1999)	Process
I20	Increased susceptibility (IS)	High genetic diversity increases the chance to defend against enemies, and therefore to invade novel environments.	Colautti et al. (2004)	Process
*I21*	*Invasional meltdown (IM)*	*The presence of alien species in an ecosystem increases the probability of invasion by additional species*.	*Simberloff and Von Holle (* *1999* *)*, *Sax et al. (* *2007* *)*	*Pattern (Process)*
*I22*	*Island susceptibility hypothesis (ISH)*	*Islands are more susceptive to biological invasions than are mainland*.	*Jeschke (* *2008* *)*, *Moser et al. (* *2018* *)*	*Pattern (Process)*
I23	Limiting similarity (LS)	The invasion success of alien species is high if their niche highly differs from that of native species, and it is low if they are similar to that of native species.	MacArthur and Levins (1967)	Process
I24	Missed mutualisms (MM) / co‐introduction	The absence of mutualist species in the introduced environment decreases the probability of invasion by an alien species.	Richardson et al. (2000), Colautti et al. (2004), Mitchell et al. (2006)	Process
I25	New associations (NAS)	Alien and native species can have novel positive or negative interactions, therefore influencing the probability of alien species to establish.	Colautti et al. (2004)	Process
I26	Novel weapons (NW)	Alien species possessing a trait that is new to native species and affects them negatively gives alien species a competitive advantage.	Callaway and Ridenour (2004)	Process
I27	Opportunity windows (OW; fluctuating resources)	Like the empty niche, but niche availability fluctuates spatially and temporally and alien species can only invade at specific places and times.	Johnstone (1986)	Process
*I28*	*Phenotypic plasticity (PH)*	*The ability of an alien species to change its phenotype to increase its fitness in a novel environment increases the probability to invade such environment*.	*Baker (* *1965* *)*, *Richards et al. (* *2006* *)*	*Trait‐based*
I29	Propagule pressure (PP)	High propagule pressure increases the chance of an alien species to invade through sheer numbers.	Lockwood et al. (2005)	Process
*I30*	*Reckless invader (RI)*	*The invasion performance of an alien species can vary, rapidly increasing its population shortly after introduction followed by a decrease in population and potentially extinction due to various reasons*.	*Simberloff and Gibbons (* *2004* *)*	*Pattern*
I31	Resource‐enemy release (RER)	Similar to the enemy release hypothesis, but assumes that invasion success is then maximized when resources are high.	Blumenthal (2006)	Process
I32	Specialist‐generalist (SG)	Enemies present in the introduced range must be specialist, and therefore less likely to affect alien species with which they have not coevolved, whereas mutualists should be generalists, to benefit alien species.	Callaway et al. (2004)	Process
*I33*	*Tens rule (TEN)*	*At every step of the invasion process, about 10% of alien species progress to the next step (Introduced, Established, Invasive)*.	*Williamson (* *1996* *)*, *Williamson and Brown (* *1986* *)*, *Jeschke and Pyšek (* *2018* *)*	*Pattern*

Notes

Italics denotes models that are not process based. Some models are considered to be pattern‐based under the strict characterization scheme, but process‐based under the inclusive characterization only. These models are classified as process‐based between parenthesis.

## Methods

### Elicitation approach

To combine community and invasion ecology into a process‐based framework, we followed an expert elicitation process during a three‐day workshop involving the co‐authors of this article, whose expertise span community and invasion ecology and a wide range of taxa. Expert elicitation is a formal procedure for obtaining and combining expert judgements, which comprises three stages: preparation, elicitation, and synthesis (Gregory et al. 2012). The sets of models and processes were collated by a core group during the preparation phase. During the elicitation phase, the community ecology and invasion models, and their underlying processes, were first discussed collectively. The workshop participants were then divided into three groups, set up to distribute the suite of expertise, to characterize the models by processes (Appendix S1, Appendix S2: Tables S1, S2, Appendix S3: Tables S1, S2). The outputs from the three groups were then organized, compared, and discussed during the synthesis phase. A second collective elicitation phase took place to match community and invasion models, and the output was synthesized and discussed. A final synthesis phase was performed by the core group to combine all results and produce a single output for the model characterization by processes and the matching of community and invasion models. General consistency in the outputs of the different working groups, many of whom had not worked together previously, during the elicitation process, indicates that despite some unavoidable degree of subjectivity for such an exercise, the synthetic results presented here reflect general and robust trends (see Appendix S1 for additional details about the elicitation processes).

### The process‐based framework

In light of our expert elicitation, we propose a framework in which community and invasion models are described according to a combination of any of the following six constituent processes: dispersal (including propagule and colonization pressure), abiotic interactions (more exactly, interactions between individuals and the abiotic environment), within‐guild biotic interactions, cross‐guild biotic interactions, ecological drift, and genetic changes (to emphasize and enable comparisons based on the stages preceding speciation; see Box 1 for definitions). This is an elaboration of Vellend’s (2016) conceptual framework originally based on four high‐level processes, namely dispersal, drift, selection, and speciation, which focuses primarily on horizontal ecological communities (i.e., species in a single trophic guild; see Vellend 2016: Chapter 2.1.1).

**Fig. 1:**
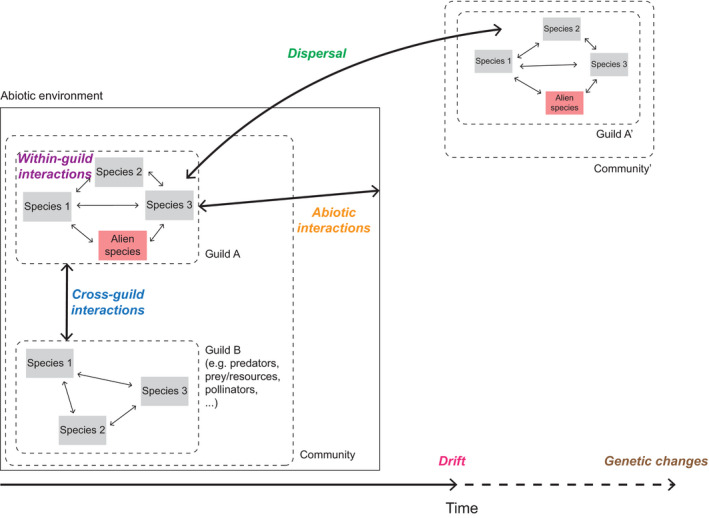
Depiction of the role of the six constituent processes (in colored italics) of the framework for determining the fate of an ecological community invaded by an alien species (red square). Cross‐guild interactions can occur with multiple other communities belonging to different trophic levels.

**Box 1. Definitions of the six constituent processes of the process‐based framework (Fig. 1)**

**Dispersal:** process encompassing the movement of organisms across space (Vellend 2016) and the propagule pressure (the number of individuals released times the number of release events; Lockwood et al. 2005), for example, seed quantity and dispersal. It is extended in this framework to encompass colonization pressure, that is, the number of species introduced or released to a single location (Lockwood et al. 2009).

**Abiotic interactions:** process representing interactions between individuals and the abiotic environment, whereby changes in the abiotic environment influence the fitness or performance of individuals from a particular species or with specific traits (Catford et al. 2009), for example, C4 plants are more adapted to warm temperatures.

**Within‐guild biotic interactions:** process whereby changes in the composition of species within the same trophic level influence the fitness or the performance of individuals from a particular species or with specific traits (Vellend 2016), for example, competition.

**Cross‐guild biotic interactions:** process whereby changes in the composition of species belonging to different trophic levels influence the fitness of individuals from a particular species or with specific traits (Vellend 2016), for example, predation.

**Drift:** any process potentially resulting in random fluctuations of species abundance, for example, disturbance (Vellend 2016).

**Genetic changes:** process based on changes in the genes of individuals, that can lead to the adaptation of a species to a given environment by modifying its traits (Keller and Taylor 2008), but also to speciation and the creation of additional species with different genomes (Vellend 2016), for example, sympatric speciation.

To describe how biotic and abiotic factors affect species’ ability to persist in an environment, we use “interactions” instead of Vellend’s “selection” to avoid confusion with population genetics terminology. In addition, following Catford et al. (2009), we split the interaction component into abiotic and biotic interactions, which can have distinct influences on species performance in communities (see also Thompson et al. 2020). Trophic interactions can be a frequent and strong driver of dynamics in meta‐communities (e.g., Guzman et al. 2019), as well as in enabling alien species to invade communities (Hui and Richardson 2017). We accordingly separated biotic interactions into within‐guild (e.g., competition, facilitation) and cross‐guild (e.g., predation, parasitism) biotic interactions (Fig. 1). In our framework, the dispersal process is associated with propagule and colonization pressure (from both external sources and established populations) because these two fundamental concepts in invasion ecology also contribute to the movement of propagules and species. In our formulation, drift includes any process potentially resulting in random fluctuations of species abundance, such as random disturbance, and therefore potentially generating stochasticity in species composition. Finally, instead of “speciation,” we use “genetic changes” in our framework; this encompasses speciation and microevolutionary processes such as local adaptation and genetic drift effects. Using these six processes provides a compromise between discriminatory power between models (six processes can be combined into 2^6^−1 = 63 different combinations) and the synthetic capacity necessary to integrate community and post‐introduction invasion ecology.

### Process‐based classification of community ecology and invasion models

We identified 32 and 33 main community and invasion models, respectively. Each model was first characterized as either process‐based or pattern‐based (with the exception of a few specific models that rely on predictions of statistical properties of the system or are trait‐based), using a definition of the model obtained from the literature (Tables 1, 2). Each process‐based model was characterized by a combination of the six processes described above (see Appendix S1 for details on the criteria used for the characterization, and Appendix S2: Tables S1 and S2, for details on why each process was considered to characterize a specific model under the two schemes). Some processes are strictly pattern‐based, but process‐based explanations have been proposed to explain these patterns, and may be considered as such under a more inclusive characterization scheme. Characterization was therefore also carried out for these models (Appendix S3: Tables S1, S2) to assess robustness of our results to vagueness and ambiguities in some of the definitions (Latombe et al. 2019). Hereafter, including these pattern‐based models in the analyses is referred to as the “inclusive characterization” scheme, as opposed to the original “strict characterization” only including purely process‐based models. We argue that the resulting set of models is representative of what has been encompassed by models, hypotheses, and theories in the two fields (e.g., Table 5.1 in Vellend 2016; also see Leibold and Chase 2017, Catford et al. 2009), although we acknowledge that one could find many additional models (e.g., Palmer 1994). Importantly, to characterize invasion models, we only considered the processes occurring after a species has become part of the pool of species already introduced in a novel environment (or susceptible to be introduced, for example the species in a region from which many goods are imported). By doing so, we can compare community and invasion models at the same spatiotemporal scale. In particular, coevolution of species in the native range underlies many invasion models (e.g., enemy release, ER, I12; missed mutualism, MM, I24), which assume that coevolved species are generally absent from the novel environment, often providing an advantage to the introduced species. However, in our framework, we do not consider that these models are characterized by genetic changes, since coevolution occurred prior to species introduction. Post‐introduction, these models are instead characterized by interaction processes. This is different from other models (e.g., evolution of increased competitive ability, EICA, I14) that rely on post‐introduction rapid genetic changes to explain invasion success and were therefore characterized by genetic changes in our framework. We then clustered the community and invasion models based on the combinations of processes that characterize them and explored which combinations of processes were the most represented in the two fields.

Under the strict characterization, three out of the 32 community models identified could not be characterized by any of the six processes and were thus excluded (Appendix S2: Table S1). Two of them (priority effects, PE, C21 and multiple stable equilibria. MSE, C17) are pattern‐ rather than process‐based (see Appendix S1 for details on criteria). In other words, they are model‐based outcomes and can be generated by multiple processes independently. Priority effects states that an initial colonizer will affect the establishment of other species, potentially facilitating some and inhibiting others. This effect is a pattern that can result from competition or predator–prey interactions, for example, modulated by frequency dependence (Fukami 2010, 2015). Within‐ and cross‐guild interactions were therefore considered to characterize the model in the inclusive characterization (Appendices S1, S3). Multiple stable equilibria posits that alternative equilibria can result from feedbacks between different possible processes (Scheffer 2009), that is, from priority effects. It was therefore considered pattern‐based using the two types of characterization. The maximum entropy theory of ecology, METE, C16, could not be characterized by the processes for a different reason: It represents a statistical property of the system leading to least‐biased predictions (Harte and Newman 2014) that cannot be pinned to any of the processes described here and therefore falls out of the scope of the present work.

Under the strict characterization, 23 of the 33 invasion models could be classified according to the six processes defined above (28 under the inclusive characterization; Appendix S2: Table S2; Appendix S3: Table S2). Eight models were based on patterns that could be implicitly explained by different processes independently (Gaertner et al. 2014): biotic acceptance (BA, I2; considered as process‐based under the inclusive characterization), biotic resistance (BR, I4; considered as process‐based under the inclusive characterization), disturbance (DS, I6; considered as process‐based under the inclusive characterization), human commensalism (HC, I18), invasional meltdown (IM, I21; considered as process‐based under the inclusive characterization), island susceptibility hypothesis (ISH, I22; considered as process‐based under the inclusive characterization), reckless invader (RI, I30), and tens rule (TEN, I33). The remaining two models, ideal weed (IW, I18) and phenotypic plasticity (PH, I28), are based on neither specific processes nor population patterns. Rather, these two models relate the invasion success of a species to its specific functional traits or its capacity to change its traits. Therefore, they refer to more fundamental mechanisms that may enable a species to invade using different propagule pressure or interaction processes independently. In other words, these two models suggest that any trait giving an advantage under one of the processes will facilitate invasion; their aim is not to explain how processes can facilitate invasion. It is important to note that by removing these pattern‐based invasion models (also those mentioned community models) from the classification does not mean that we ignored them completely, even under the strict characterization. Instead, we discuss how they can be incorporated in the framework and related to the process‐based models in the Discussion.

### Correspondence between specific community and invasion models

We matched each community model to a set of corresponding invasion models based on the similarity of the processes involved, identified in the previous step. Because we only used six processes to keep a synthetic capacity in the framework (therefore sometimes overlooking subtleties in how the processes were conceptualized in specific community and invasion models), we relied on the careful examination of how these processes were defined in each model, rather than only using an oversimplified criterion on the number of shared processes (but see the Discussion below for details on how the framework could evolve to capture more accurately the specificities of the models). We therefore compared the definitions of all community and invasion models, and we explain how the processes that characterize them in the framework are related (some models in one field being special cases of a model in the other, and some models being almost equivalent in the two fields, for example, see Appendix S2: Table S3). As explained above, we only considered post‐introduction processes for invasion models.

**Fig. 2:**
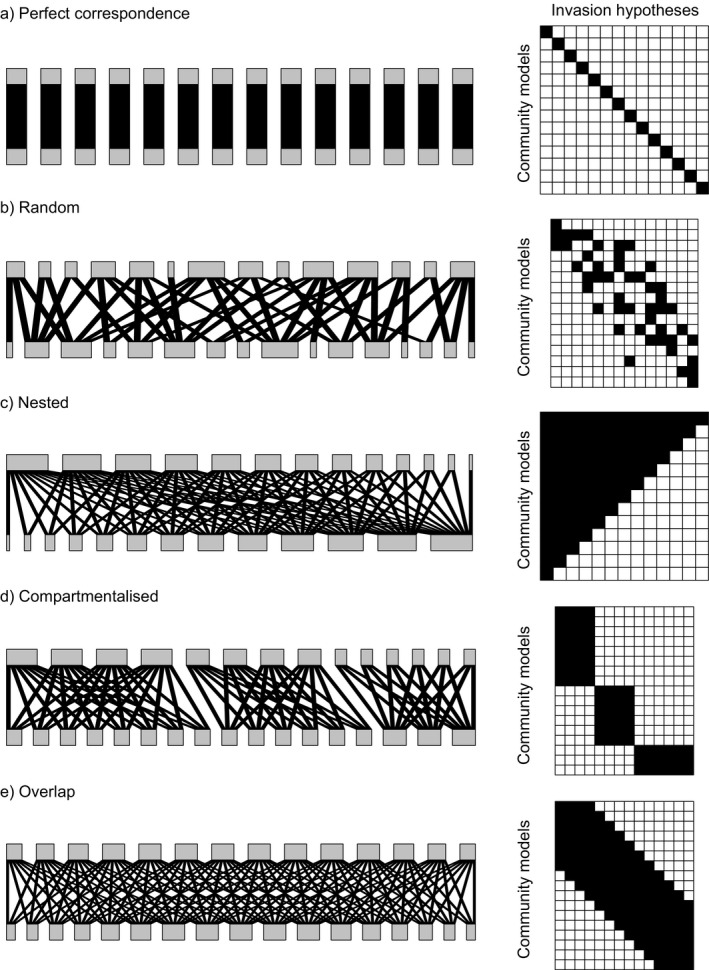
Five archetypes that can characterize the relationship between community and invasion models, representing different levels of alignment or misalignment. The perfect correspondence and nested archetypes require the same number of community and invasion models, whereas the others do not.

The alignment of community and invasion models was visualized as a bipartite matrix, and the relationship between two fields was analyzed using tools from network theory. We envisaged five possible archetypes defining how community models can be related to invasion models, therefore representing different levels of alignment or misalignment between models in the two fields (Fig. 2). With perfect correspondence, each community model would be related to only one invasion model, and reciprocally (Fig. 2a). This archetype would only be possible if no overlap existed between models within a field, making both fields perfectly aligned. A random configuration would indicate that both fields do not correspond well, with a lot of overlap between the models both within and across the two fields (Fig. 2b). In a perfectly nested scenario, each field would have one overarching model that can be decomposed into more specific models (Fig. 2c). In other words, nestedness would occur when a combination of multiple processes encompasses a subset of these processes (e.g., dispersal is nested in the combination of dispersal and any other processes). A compartmentalized configuration would be a more realistic version of perfect correspondence, with community and invasion models grouped into several clusters. This would illustrate that the two fields follow a similar logic, but with some overlap between models within each field (Fig. 2d). In particular, greater horizontal than vertical width of clusters would indicate that a community model is considered under various perspectives in invasion ecology, and reciprocally. Finally, the overlap situation can also be seen as an extension of perfect correspondence, in which one community model can be related to several invasion models, and reciprocally, in a non‐random fashion consistent with the processes that define them (Fig. 2e). Overlap would occur when two different combinations of processes share a common process (e.g., the combination of dispersal and abiotic niche interactions would overlap the combination of dispersal and within‐guild niche interactions). The width of the diagonal would be inversely proportional to the coherence within and across fields, reflecting different research focuses of each field.

We assessed whether the observed bipartite matrix was more nested than by chance (i.e., if it corresponded to the nested archetype) by comparing the nestedness indices of the observed to that of 999 randomized matrices (randomizing all possible associations). A modularity analysis using the Dormann‐Strauss algorithm (using function computeModules form the bipartite R package V2.11; Dormann et al. 2008) was used to identify clusters under the compartmentalized archetype. Since the number and nature of the modules can vary slightly across different runs of the algorithm, we ran 100 replicates of the algorithm and selected the output with the highest likelihood. The likelihood of the observed modularity was then compared to that of the 999 randomized matrices to assess if the observed matrix was more compartmentalized than by chance. All analyses were performed using R 3.6.1 (R Core Team 2019).

## Results

### Process‐based classification of community models

**Fig. 3:**
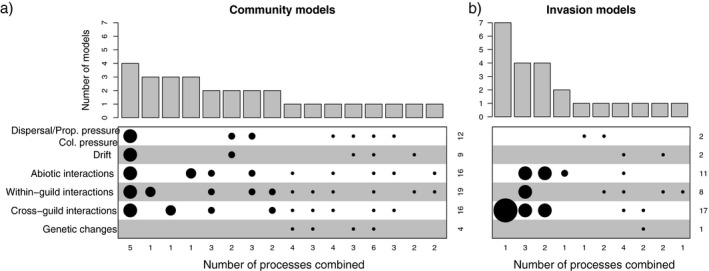
Summary of combinations of processes characterizing (a) the community models (*n* = 29) and (b) the invasion models (*n* = 23; see Appendix S2: Tables S1 and S2 for details on process combinations). The lower plots indicate the combinations of processes that were identified, ordered by the number of models characterized by a specific combination (processes combined in a model are represented by the circles in a single column, and the number of combined processes is also indicated by the numbers at the bottom of the plots). The numbers on the right of the graphs represent the number of models that include each process. The size of the circles and the upper bar plot both indicate this number (the bar plot was used to better visualize the skewness of the distributions).

Sixteen different combinations of processes were identified to characterize the 29 (30 under the inclusive characterization) process‐based community models. The models combined 2.62 processes on average (2.6 under the under the inclusive characterization). The most common combination of processes (included in four models) included all processes except genetic changes. The second most common combinations of processes included different combinations of the three interaction‐based processes (each of these different combinations being included in three models; Fig. 3a). The within‐guild biotic interaction process was the most common process, characterizing 19 models. Overall, biotic interactions (within‐ and between‐guild combined) characterized 23 out of the 29 models, and interactions (biotic and abiotic combined) characterized 26 (~70%) models. In contrast, genetic changes are the least represented process, characterizing only four of the 29 models. Results were qualitatively similar under the inclusive characterization (Appendix S3).

### Process‐based classification of invasion models

Ten combinations of processes were identified to characterize the 23 invasion models (12 combinations for the 28 models under the inclusive characterization; Appendix S3). The models combined 1.78 processes on average (1.86 under the under the inclusive characterization). By far, the most common process characterizing invasion models is cross‐guild interactions. Seven models relied on this single process alone, and 10 other models included it as one of their driving processes (Fig. 3b; 14 models included it as one of their driving processes under the inclusive characterization Appendix S3: Fig. S1). The three interaction processes unequivocally dominated invasion models under both the strict and inclusive characterizations, and dispersal, drift and genetic changes only characterized five invasion models.

### Correspondence between specific community ecology and invasion models

**Fig. 4:**
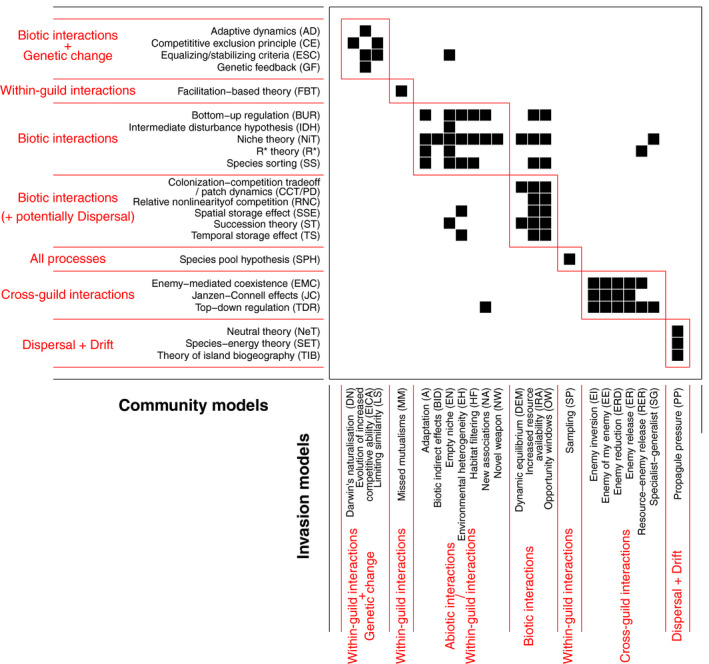
Relationship between community (rows) and invasion (columns) models presented as a bipartite network. The modules were identified using the Dormann‐Strauss algorithm. The main processes characterizing each module are indicated in red: / indicates that at least one process characterizes the models, whereas + indicates that the processes are combined in the models.

The relationship between community and invasion models most closely reflected a mixture of three of the possible five archetypes, nested, compartmentalized, overlap, although there were some elements of the perfect correspondence and randomness archetypes (Figs. 2, 4, Appendix S2: Fig. S1, Appendix S3: Fig. S2). The randomization algorithm revealed a relatively high level of nestedness: The observed nestedness was higher than for 97.7% of the randomized matrices (99.9% for the inclusive characterization). This can be expected when some umbrella theories encompass more specific models (Appendix S2: Fig. S1, Appendix S3: Fig. S3). In particular, the more encompassing community models are niche theory (NiT, C20), bottom‐up regulation (BUR, C2), top‐down regulation (TDR, C32) and species sorting (C26; i.e., those based on combinations of interaction processes). The most encompassing invasion models are Increased resource availability (IRA, I19) and opportunity windows (OW, I27), followed by the empty niche model (EN, I8), which all cover aspects related to interactions, temporal variability, and availability of niches.

The modularity analysis identified seven modules (the observed likelihood of the modularity was higher than for 99.8% of the randomized matrices), therefore partly reflecting the compartmentalized archetype (Fig. 4). The bi‐adjacency matrix shows four modules grouping between three and seven community or invasion models each. The models of these modules are based predominantly on combinations of interaction processes. These modules are nonetheless not perfectly defined, as there are some associations close to the diagonals but not belonging to any module. Therefore, they also contain characteristics of the overlap or random archetypes, indicating some lack of coherence within and between these models in the two fields. There are also three smaller modules, which tend toward the perfect correspondence archetype. These smaller modules are based on dispersal, genetic changes, and specific aspects of niche theory (mostly positive interactions of mutualism), or on the effect of species on the environment rather than the opposite. Results are similar using the inclusive characterization (the observed likelihood of the modularity was higher than for 99.9% of the randomized matrices), but the bi‐adjacency matrix shows more dispersed associations of community and invasion models, characteristic of the random archetype (Appendix S3: Fig. S2).

Four process‐based community models (stochastic niche theory, SN, C28; neutral‐niche continuum, NNC, C19; community assembly phase space, CAPS, C4; and intransitive competition, IC, C13) were not related to any invasion model. The first three models (SN, NNC, and CAPS) are all defined by a combination of dispersal, drift, and the three interaction processes. IC considers within‐guild interactions from a multi‐species perspective. One process‐based invasion model, increased susceptibility, IS, I20, was not related to any community model. IS is based on interaction processes, but relates them to genetic diversity (but not to the process of genetic changes), which none of the community models listed here did explicitly.

## Discussion

### Differences between the processes addressed by invasion and community ecology

Characterizing and matching invasion and community models according to their underlying processes using our framework highlights important differences in research focus in the two fields. About a quarter of the invasion models considered rely on the classification of invasion patterns under the strict characterization. Process‐based invasion ecology models also appear to more often consider the role of single mechanisms in isolation, as shown by the low number of processes in combinations. The smaller number of multi‐process models in invasion ecology (Fig. 2b) is consistent with the search for case‐specific explanations of biological invasions integrating information about species biology and ecosystem characteristics, that is, invasion syndromes (Kueffer et al. 2013, Perkins and Nowak 2013, Novoa et al. 2020). Invasion ecology indeed often relies on observational approaches (see Fig. 17.3 in Jeschke and Heger 2018) allowing only limited control on the conditions of invasion. These approaches are therefore designed to investigate specific processes (see Jeschke and Heger 2018 for a synthesis of support or rejection of different models based on such approaches in the literature). In contrast, the larger number of processes used in combination in the community models reflects the fact that community ecology has strived for a more overarching, mechanistic perspective that emphasizes how the interplay of multiple processes can address a wide range of questions on the generation, dynamics, maintenance, and evolution of communities over a wide range of temporal and spatial scales (Gravel et al. 2006, Latombe et al. 2015, Vellend 2016, Leibold and Chase 2017).

Differences in the number of processes considered by community and invasion models can be explained by the different level of emphasis on interaction processes in the two fields. The predominance of interaction processes (especially cross‐guild interactions) in the list of invasion models has led us to identify a number of overlapping models, and therefore a highly skewed distribution of processes across invasion models (Fig. 3b), which could be a source of ambiguities (Latombe et al. 2019). For example, the enemy inversion (EI), the enemy of my enemy (EE), the enemy reduction (ERD), and the enemy release (ER) models (I9‐I12) are all variations of the same cross‐guild interaction process (although such models can be further distinguished and related to each other using a hierarchy of hypotheses; Jeschke et al. 2012, Jeschke and Heger 2018). Although the distribution of processes is less skewed for community models (Fig. 3a), interaction processes are also the focus of a number of community models, which can be explained by the fact that interaction processes also dominated community ecology for decades (e.g., reviewed in Leibold 1995, Chase and Leibold 2003). Within‐guild processes are nonetheless predominant, indicating a focus on horizontal communities, consistent with Vellend’s (2016) original framework. Consistently, the biggest modules in the bi‐adjacency matrix (Fig. 4) contain community and invasion models based predominantly on combinations of all interaction processes. Due to the high degree of attention they received historically, it is not surprising that interaction processes have been combined in many different ways, resulting in a limited degree of coherence between models based on these processes within and across the two fields.

More recent depictions of community models (e.g., SN, Tilman 1994; NNC, Gravel et al. 2006; CAPS, Latombe et al. 2015), however, spurred on by Hubbell’s (2001) neutral theory which emphasized the role of dispersal and stochasticity, provide a more balanced perspective, and recognize the interplay of multiple processes, rather than considering independent processes in isolation (Vellend 2016, Leibold and Chase 2017). In contrast, few invasion models consider post‐introduction processes other than interaction processes, resulting in fewer combinations of processes overall. Most invasion studies on dispersal focus on the human‐mediated introduction/invasion pathways (e.g., Wilson et al. 2009). However, it has also been shown that different dispersal kernels (e.g., Hui et al. 2012), and especially the presence or absence of long‐distance dispersal (Berthouly‐Salazar et al. 2013), are crucial for determining the range expansion of alien species in novel environments (Kot et al. 1996, McGeoch and Latombe 2016). Given the importance of feedback between dispersal and interactions for explaining community assembly (Latombe et al. 2015), and the role of spatial and temporal correlations of stochasticity in population size and growth in boosting invasion performance (Cuddington and Hastings 2016, Hui et al. 2017), combinations of neutral and interaction processes will likely reveal unexpected trajectories for both the invaders and the structure of the recipient community, even for single‐species invasions. This also applies to the combination of these processes with genetic changes, as rapid evolutionary changes in introduced species have been shown to be quite commonly associated with invasion success (Whitney and Gabler 2008).

Lawton (1996) wrote with reference to patterns in community ecology: “Too often, ecologists seem obsessed with finding a single explanation for some process or pattern of interest.” Community ecology has, however, recently transitioned toward a more comprehensive perspective that embraces the interplay between multiple processes. There are several possible reasons why invasion ecology often considers the role of specific processes in isolation to explain biological invasions. First, invasion models tend to have a narrower scope (exploring factors that mediate survival and establishment of a particular introduced species in a novel environment; Pysek et al. 2020) compared to community models (whose scope range from the generation and dynamics to the maintenance and evolution of communities). More importantly, invasion ecology has only started to develop as a field more recently (Vaz et al. 2017). Searches on Web of Science with the keywords “community ecology” and “invasion ecology” as topics return articles dating back to 1914 and 1986, respectively. It is therefore possible that the lists of models used here, which have similar lengths, may overlook overlaps between community models that may have existed when the field was younger. This list also likely underestimates the number of early community models focusing on the identification of patterns, such as the mathematical formulation of species‐area relationships (Connor and McCoy 1979) or species abundance distributions (Williamson and Gaston 2005). This is actually good news for invasion ecology, as it would indicate that the field can benefit from the long history of community models to develop further from a mechanistic perspective and produce a coherent synergy between the two fields, as we elucidate below.

### Toward a stronger synergy between invasion and community ecology

Fitting the process‐based framework presented here to existing models, theories, and hypotheses is useful to obtain a much‐needed coherent and synthetic picture and an overarching view of community and invasion ecology. Because of the small number of processes considered simultaneously by invasion models, we argue that we should move toward emphasizing a process‐based invasion ecology, to complement the experimental search for specific reasons to explain successful biological invasion events.

Attempts to reconcile community and invasion ecology have often focused on specific interaction processes between one alien species and a native community. Shea and Chesson (2002) introduced the concept of niche opportunity, which encompasses the different interaction processes of our framework. In their this framework, niche opportunities allow an invading population to have a positive growth rate through access to resources or decrease in natural enemies. MacDougall et al. (2009) extended this concept by building on the perspective of equalizing vs stabilizing mechanisms as proposed by Chesson (2000*a*). Wolkovich and Cleland (2011) showed how phenology can also provide niche opportunities. Pearson et al. (2018) further incorporated dispersal processes by building on the similarity between the dispersal, abiotic, and biotic ecological filters from community ecology (e.g., Stokes and Archer 2010) and invasion ecology (Catford et al. 2009). Although each of these frameworks has included several of the six processes described in this paper, they were considered either separately, or additively, not in a truly interactive fashion considering feedbacks and complex outcomes, as explored by community ecology and highlighted by our framework.

A truly mechanistic perspective of biological invasions would follow the direction taken by more recent community ecology models (e.g., Gravel et al. 2006, Latombe et al. 2015, Leibold and Chase 2017) by exploring how different combinations and feedbacks between the processes described in this framework generate different community and invasion patterns. It would then be possible to generate hypotheses that can be systematically tested through experiments or field observations. This approach would enable invasion and community ecology to advance simultaneously. This would help encourage further research on multi‐species interactions in invasion ecology. Such a whole system approach will enable us to achieve a more complete picture of biological invasions (Gurevitch et al. 2011), to understand and potentially predict the fate of invaded communities, including the trajectories leading to regime shifts (Gaertner et al. 2014) and the dynamics of thresholds between historical, hybrid, and novel ecosystems (Hobbs et al. 2014). This will in turn contribute to improve our understanding of community assembly and structure.

To develop such a mechanistic, process‐based approach to invasion ecology, future work should clarify the relationship between process‐ and pattern‐based invasion models. This framework should establish the relationship between invasion patterns and different combinations of processes. Patterns generated by the same sets of processes could then also be related to each other (Appendix S2: Fig. S2). This would also enable us to clearly define nestedness and partial overlap between models, as both metrics are defined based on process similarity. Using this approach will also enable us to remove potential ambiguities when pairing community ecology and invasion models.

We acknowledge that our six‐process framework may evolve to capture more accurately the specificities of the models. This is why it was designed in a hierarchical fashion from Vellend’s (2016) initial four high‐level processes, which already captured the essence of the relevant processes. For example, we have not characterized biotic interactions as positive (mutualistic) or negative (antagonistic), although both kinds have been argued to be important drivers of species assembly and coexistence in community ecology, and of invasion success in invasion biology (e.g., Francis and Read 1995, Christian 2001, Colautti et al. 2004, Traveset and Richardson 2020). Other mechanisms, such as frequency dependence, which can apply to different processes and are integral parts of some models (e.g., priority effects), could also be considered in parallel to this framework. While restricting our framework to six processes allowed for generality and a broad, synthetic perspective across community and invasion ecology, considering additional processes may reveal complex and unexpected behaviors in modeled invaded communities.

Finally, it is important to explicitly incorporate spatial and temporal scales when expanding this process‐based framework. The processes defined here, as those on which they are based (Catford et al. 2009, Vellend 2016), are not restricted to any particular scale. Rather, as scale is important to detect ecological patterns such as changes in species richness and turnover over space and time (Chase et al. 2018), it may change the perspective on the importance of each process at play (Chase 2014, Viana and Chase 2019). For example, competition may only be detected at fine spatial scales (e.g., between adjacent fruiting plants), whereas cross‐guild interactions with frugivorous birds dispersing seeds would occur at a much larger scale. Environmental heterogeneity also varies across scales, changing our perception of the importance of related processes. The relevant scales therefore depend on how the involved taxa perceive and are affected by, the different processes over specific spatial and temporal scales (Theoharides and Dukes 2007, McGill 2010). This process‐based framework, like those on which it is based, can therefore offer a bridge between multiple scales (Vellend 2016).

## Conclusion

We have presented a mechanistic framework to classify both community and invasion models, using combinations of six different processes: dispersal, drift, abiotic interactions, within‐guild interactions, cross‐guild interactions, and genetic changes. Characterizing models according to these processes allowed us to avoid biases and gaps from overly focusing on specific processes. The classification of representative models from the two fields following this framework and their comparison using a novel method based on network theory has helped not only to provide a synthesis of representative models in the two fields, but also to identify differences and overlaps between them. This enables us to identify where there may be scope to increase coherence both within (as Catford et al. 2009, Vellend 2016) and across these fields in the future. In particular, it shows that concepts in invasion ecology tend to focus on the identification of specific processes, whereas community ecology has transitioned to explore how different combinations of multiple processes can provide a more mechanistic understanding of a whole suite of patterns. We hope that the bridge developed in this paper will help to advance both fields concurrently following a process‐based approach generating hypotheses to be validated experimentally. Using perspectives from one field to investigate questions in the other may create an integrative perspective in ecology that is still lacking (Rosindell et al. 2015, Courchamp et al. 2017, Pearson et al. 2018), advancing a more predictive ecology that is sorely needed in a rapidly changing world.

## Supporting information

Appendix S1

Appendix S2

Appendix S3
