# Supplementary material for: Mechanistic reconciliation of community and invasion ecology
Source: Ecosphere. 2021 Feb 10;12(2):e03359. doi: 10.1002/ecs2.3359 (PMC8647914; doi:10.1002/ecs2.3359)
Supplement: Supplementary file 1 — Appendix S1 [file ECS2-12-e03359-s001.pdf]

Supporting Information.

Latombe G, Richardson DM, McGeoch MA, Altwegg R, Catford JA, Chase JM, Courchamp F, Esler KJ, Jeschke JM, Landi P, Measey J, Midgley GF, Minoarivelo HO, Rodger JA, Hui C. Mechanistic reconciliation of community and invasion ecology. *Ecosphere.*

Appendix S1. Details and criteria for the application of the framework

Selection of invasion models

Enders et al. (2018) list 33 models, but after examining the literature (Crawley et al. 1999, Alpert 2006), we considered that Global competition and Sampling models could not be distinguished from one another, at least with respect to the processes invoked in the classification framework, and therefore merged them into a single model (H16 in Table 2). To these 32 models, we added Phenotypic plasticity.

Criteria for process characterization

A community or an invasion model was considered to involve one of the six constituent processes included in the framework if (i) the process is specifically mentioned in its definition, and (ii) it assumes that the inclusion or exclusion of the process (or change in its strength) can result in non-trivial changes in the community or the invasion patterns predicted by the model. As it is shown below, some models are defined from observed community patterns or patterns of species distribution. These patterns may be generated by different processes independently (e.g. the same pattern may be generated by either dispersal or a interaction process) and no process complies with criterion (i). Such models were discarded from the analyses. In contrast, some

models are general and encompass multiple lower-level processes, and are thus defined by a higher-level process. For example, niche theory is defined by any combination of interaction processes in a non-exclusive fashion and is characterized by all possible lower-level processes that fall within the higher-level process (e.g. niche theory, includes abiotic, within-guild and cross-guild biotic interaction processes). The definition is still process-based (and the lower-level processes are not exclusive from each other) and is considered to comply with criterion (i), contrary to the pattern-based definitions. Such models were therefore considered in the analyses.

We acknowledge that many models emphasize the role of one particular process, but remain non-exclusive regarding the roles of other processes. We considered that not mentioning a process when defining a model implies that the incidence and strength of the process either varies negligibly across spatial and temporal scales, or has a negligible impact on community structure or on invasion success despite potentially varying. Such a process therefore does not comply with criterion (ii). Finally, biotic interactions (within- or cross-guild) are considered to exist if the presence/absence of individuals from another species changes the community or the invasion pattern, even if the individuals of the two species do not interact directly (e.g. limiting similarity and trait comparisons between pairs of natives and aliens).

This classification exercise is challenging, as most models are defined according to general principles that contain some vagueness and ambiguity (Latombe et al. 2019). To combat this, we provided clear justifications and criteria for each of the choices when trying to identify a set of processes characterizing each model. We nonetheless acknowledge that this has involved some degree of subjectivity. For example, the increased resource availability (IRA, H19) model was

assumed to be characterized by abiotic interactions only. However, the increase in resource may be caused by the presence of certain species, such as nitrogen-fixing invasive plants, in which case IRA would be confounded with invasional meltdown (H21) for this specific situation. Similarly, disturbances can reduce abundance of native plants and therefore resource uptake, in which case IRA and Disturbance (H6) would also be confounded. Such subjectivity and the resulting uncertainties also lead to little agreement between scientists when asked to relate invasion models to each other based on their similarity or dissimilarity (Enders et al. 2018). As a result, we could expect another team of experts to generate slightly different results if they were to perform the same exercise here, and readers may disagree with the inclusion or exclusion of some of the processes in the different models, as exemplified by the fact that we aggregated the Global competition and the Sampling invasion models, contrary to previous studies (Catford et al. 2009, Enders et al. 2018). However, general consistency in the outputs of the different working groups, many of whom had not worked together previously, during the elicitation process, indicates that the synthetic results presented here reflect general and robust trends, despite some unavoidable degree of subjectivity for such an exercise.

References

Alpert, P. 2006. The advantages and disadvantages of being introduced. Biological Invasions 8:1523–1534.

Catford, J. A., R. Jansson, and C. Nilsson. 2009. Reducing redundancy in invasion ecology by integrating hypotheses into a single theoretical framework. Diversity and Distributions 15:22–40.

Crawley, M. J., S. L. Brown, M. S. Heard, and G. R. Edwards. 1999. Invasion-resistance in experimental grassland communities: species richness or species identity? Ecology Letters 2:140–148.

Enders, M., M. Hütt, and J. M. Jeschke. 2018. Drawing a map of invasion biology based on a network of hypotheses. Ecosphere 9:e02146.

Latombe, G., S. Canavan, H. Hirsch, C. Hui, S. Kumschick, M. M. Nsikani, L. J. Potgieter, T. B. Robinson, W. Saul, and S. C. Turner. 2019. A four-component classification of uncertainties in biological invasions: implications for management. Ecosphere 10:e02669.
