# Supplementary material for: Mechanistic reconciliation of community and invasion ecology
Source: Ecosphere. 2021 Feb 10;12(2):e03359. doi: 10.1002/ecs2.3359 (PMC8647914; doi:10.1002/ecs2.3359)
Supplement: Supplementary file 2 — Appendix S2 [file ECS2-12-e03359-s002.pdf]

**Supporting Information.**

Latombe G, Richardson DM, McGeoch MA, Altwegg R, Catford JA, Chase JM, Courchamp F, Esler KJ, Jeschke JM, Landi P, Measey J, Midgley GF, Minoarivelo HO, Rodger JA, Hui C. Mechanistic reconciliation of community and invasion ecology. *Ecosphere.*

**Appendix S2. Supplementary tables and figures**

**Table S1.** Justification of process inclusion of each of the community models. Parts in quotes are taken from the definition provided in Table 1, making the inclusion of the process obvious.

| **ID** | **Community model** | **Reference(s)** | **Dispersal / Propagule pressure / Colonization pressure** | **Drift** | **Interactions** | | | **Genetic changes (speciation, adaptation)** |
|---|---|---|---|---|---|---|---|---|
| | | | | | **Abiotic interactions** | **Biotic interactions** | | |
| | | | | | | **Within-guild** | **Cross-Guild** | |
| C1 | Adaptive dynamics (AD) | (Fussmann et al. 2007) | | | | "driven by ecological interaction" | "driven by ecological interaction" | "Mutation limited evolution" |
| C2 | Bottom-up regulation (BUR) | (Oksanen et al. 1981, Matson and Hunter 1992) | | | | | "driven by resources (lower trophic levels)" | |

| | | | | | | | |
|---|---|---|---|---|---|---|---|
| C3 | Colonization-competition trade-off / patch dynamics (CCT/PD) | (Levins and Culver 1971) | "Good colonizers (dispersers)" | | Unsuitable abiotic conditions would make "bad competitors" | "Bad competitors" can be interpreted and directly modelled as within-guild interactions | The presence of enemies would make "bad competitors" |
| C4 | Community Assembly Phase Space (CAPS) | (Latombe et al. 2015) | Part of the model | Part of the model | Part of the model | Part of the model | Part of the model |
| C5 | Competitive exclusion principle (CE) | (Gause 1934) | | | | "Two species competing" | |
| C6 | Ecosystem engineering (EE) | (Jones et al. 1994) | | | "effects of one species on the abiotic environment" | | |
| C7 | Enemy-mediated coexistence (EMC) | (Holt et al. 1994) | | | | The relative abundance of another species determines the effect of enemies | The enemy is from another guild |
| C8 | Equalizing/stabilizing criteria (ESC) | (Chesson 2000a) | | | fitness can be determined by abiotic conditions | "niche differentiation between species" | fitness can be determined by enemies |
| C9 | Facilitation-based theory (FBT) | (Bruno et al. 2003) | | | | "positive interactions between species" (e.g. | "positive interactions between |

| | | | | | | | | |
|---|---|---|---|---|---|---|---|---|
| | | | | | | association between fungi and algae in lichens) | species" (e.g. pollination) | |
| C10 | Genetic feedback (GF) | (Pimentel 1968) | | | Included in "interaction abilities" | Included in "interaction abilities " | Included in "interaction abilities " | The change in interaction abilities occurs through genetic adaptation |
| C11 | Hump-shaped diversity-productivity hypothesis (HSDPH) | (Grime 1973) | | | | "competitive exclusion" | | |
| C12 | Intermediate disturbance hypothesis (IDH) | (Grime 1973, Connell 1978) | | "disturbance" can generate random fluctuations in species abundance | | "decreases competition" | | |
| C13 | Intransitive competition (IC) | (Gilpin 1975) | | | | "competitively superior to some and inferior to others" | | |
| C14 | Janzen-Connell effects (JC) | (Connell 1970, Janzen 1970) | | | | | "Species-specific enemies accumulate | |

| | | | | | | | | |
|---|---|---|---|---|---|---|---|---|
| | | | | | | | around adult trees" | |
| C15 | Mass effect (ME) | (Holyoak et al. 2005, Leibold and Chase 2017) | "Colonization from occupied sites" | | "to survive in a site with unfavorable environment" | | The presence of enemies would make "an unfavorable environment" | |
| C18 | Neutral theory (NeT) | (Hubbell 2001) | Part of the model | Part of the model | | | | Part of the model |
| C19 | Neutral-niche continuum (NNC) | (Gravel et al. 2006) | Part of the model | Part of the model | Part of the model | Part of the model | Part of the model | |
| C20 | Niche theory (NiT) | (Chase and Leibold 2003) | | | Included in "interaction processes" | Included in "interaction processes" | Included in "interaction processes" | |
| C22 | R* theory (R*) | (Tilman 1982) | | | "dealing with multiple resources" | The R* is compared between multiple species | | |
| C23 | Relative nonlinearity of competition (RNC) | (Armstrong and McGehee 1980) | | | "Interactions with resources" | | | |
| C24 | Spatial storage effect (Chesson 2000) | (Chesson 2000b) | The persistence (e.g. through seed banks) is possible due to propagule pressure | | The abiotic environment fluctuates spatially | "interspecific competition is greatest at low abundance" | | |

| | | | | | | | | |
|---|---|---|---|---|---|---|---|---|
| C25 | Species pool hypothesis (SPH) | (Taylor et al. 1990) | Regional and historical dispersal shapes the species pool | Regional and historical drift shapes the species pool | Regional and historical interaction processes shapes the species pool | Regional and historical interaction processes shapes the species pool | Regional and historical interaction processes shapes the species pool | Genetic changes shape the species pool |
| C26 | Species sorting (SS) | (Holyoak et al. 2005, Leibold and Chase 2017) | | | "Species differ in their fitness in different abiotic environments " | | | |
| C27 | Species-energy theory (SET) | (Wright 1983) | Dispersal and propagule pressure determine immigration | Drift is related to local species extinction | | | | |
| C28 | Stochastic niche theory (SN) | (Tilman 2004) | "Niche theory incorporating drift and propagule pressure" | "Niche theory incorporating drift and propagule pressure" | "Niche theory incorporating drift and propagule pressure" | "Niche theory incorporating drift and propagule pressure" | "Niche theory incorporating drift and propagule pressure" | |
| C29 | Succession theory (ST) | (Pickett et al. 1987) | "incorporating all processes but speciation" | "incorporating all processes but speciation" | "incorporating all processes but speciation" | "incorporating all processes but speciation" | "incorporating all processes but speciation" | |
| C30 | Temporal storage effect (TS) | (Chesson 2000b) | The persistence (e.g. through seed banks) is possible due | | The abiotic environment fluctuates temporally | "interspecific competition is greatest at low abundance" | | |

| | | | | | | | | |
|---|---|---|---|---|---|---|---|---|
| | | | to propagule pressure | | | | | |
| C31 | Theory of island biogeography (TIB) | (MacArthur and Wilson 1967) | Dispersal and propagule pressure determine immigration | Drift is related to local species extinction | | | | |
| C32 | Top-down regulation (TDR) | (Matson and Hunter 1992) | | | | | "driven by predators (higher trophic levels)" | |

**Table S2.** Justification of process inclusion of each of the invasion models. Parts in quotes are taken from the definition provided in Table 2, making the inclusion of the process obvious.

| ID | Invasion model | Reference(s) | Dispersal / Propagule pressure / Colonization pressure | Drift | Interactions | | | Genetic changes (speciation, adaptation) |
|---|---|---|---|---|---|---|---|---|
| | | | | | Abiotic interactions | Biotic interactions | | |
| | | | | | | Within-guild | Cross-Guild | |
| I1 | Adaptation (A) | (Duncan and Williams 2002) | | | Defines the "conditions in the exotic range" | | Defines the "conditions in the exotic range" | |
| I3 | Biotic indirect effects (BID) | (Callaway et al. 2004) | | | "Combinations of cross-guild and potentially abiotic processes" | | "Combinations of cross-guild and potentially abiotic processes" | |
| I5 | Darwin's naturalization (DN) | (Darwin 1859) | | | The phylogenetic proximity indicates similar interaction process, abiotic or biotic | The phylogenetic proximity indicates similar interaction process, abiotic or biotic | The phylogenetic proximity indicates similar interaction process, abiotic or biotic | |

| | | | | | | | |
|---|---|---|---|---|---|---|---|
| I7 | Dynamic equilibrium (DEM) | (Huston 1979) | | "natural fluctuations of the ecosystem" can generate random fluctuations in species abundance | | "which influences the competition of local species" | |
| I8 | Empty niche (EN) | (MacArthur 1970) | | | The "niche" is a broad concept that encompasses abiotic and biotic interactions processes | The "niche" is a broad concept that encompasses abiotic and biotic interactions processes | The "niche" is a broad concept that encompasses abiotic and biotic interactions processes |
| I9 | Enemy inversion (EI) | (Colautti et al. 2004) | | | "due to altered biotic and abiotic conditions" | "due to altered biotic and abiotic conditions" | "Introduced enemies of alien species " |
| I10 | Enemy of my enemy (EE) | (Eppinga et al. 2006) | | | | | "Introduced enemies of an alien species are more harmful" |
| I12 | Enemy reduction (ERD) | (Colautti et al. 2004) | | | | | "Enemies are less frequent in the introduced range" |

| | | | | | | | | |
|---|---|---|---|---|---|---|---|---|
| I13 | Enemy release (ER) | (Keane and Crawley 2002) | | | | | "Enemies are absent in the introduced range" | |
| I14 | Environmental heterogeneity (EVH) | (Melbourne et al. 2007) | | | "A highly heterogeneous environment" refers to abiotic conditions | | | |
| I15 | Evolution of increased competitive ability (EICA) | (Blossey and Notzold 1995) | | | | | "Release from natural enemies" | "this re-allocation is due to genetic changes" |
| I16 | Global competition (GC) – equivalent to Sampling (SP) | (Crawley et al. 1999, Alpert 2006) | "A large number of different alien species" corresponds to colonization pressure | | | "at least one of them will outcompete native species due to interaction processes" | | |
| I17 | Habitat filtering (HF) | (Darwin 1859, Melbourne et al. 2007) | | | "whose niche fits the abiotic environment" | | | |
| I20 | Increased resource availability (IRA) | (Sher and Hyatt 1999) | | | "Resource" encompasses the abiotic environment | | "Resource" encompasses the lower trophic level | |

| | | | | | | | |
|---|---|---|---|---|---|---|---|
| I21 | Increased susceptibility (IS) | (Colautti et al. 2004) | | | | | "defend against enemies" |
| I24 | Limiting similarity | (MacArthur and Levins 1967) | | | "Niche similarity" encompasses abiotic interaction processes | "Niche similarity" encompasses biotic interaction processes | "Niche similarity" encompasses biotic interaction processes |
| I25 | Missed mutualisms (MM) / co-introduction | (Colautti et al. 2004, Mitchell et al. 2006) | | | | | The mutualism usually occurs between species of different guilds (Richardson et al. 2000) |
| I26 | New associations (NAS) | (Colautti et al. 2004) | | | | | This is the counterpart of missed mutualism |
| I27 | Novel weapons (NW) | (Callaway and Ridenour 2004) | | | | The novel weapon is targeted towards competitors, such as release of chemicals through roots | |

| | | | | | | | |
|---|---|---|---|---|---|---|---|
| I28 | Opportunity windows (OW; fluctuating resources) | (Johnstone 1986) | | Drift can create a temporal opportunity | The "niche" is a broad concept that encompasses abiotic and biotic interaction processes | The "niche" is a broad concept that encompasses abiotic and biotic interaction processes | The "niche" is a broad concept that encompasses abiotic and biotic interaction processes |
| I30 | Propagule pressure (PP) | (Lockwood et al. 2005) | "Propagule pressure" | | | | |
| I32 | Resource-enemy release (RER) | (Blumenthal 2006) | | | "Resources" encompasses the abiotic environment | | Like the enemy release. Resource can also encompass a lower trophic level. |
| I33 | Specialist-generalist (SG) | (Callaway et al. 2004) | | | | | This hypothesis considers interactions with enemies and mutualists, considered to belong to different guilds. |

**Table S3.** Associations between process-based community and invasion models. The processes linking community and invasion models are indicated in bold. Shading is only used to distinguish between community models.

| Community models/theories | Invasion models | Justification |
|---|---|---|
| Adaptive dynamics (AD) | Evolution of increased competitive ability (EICA) | Once a species is introduced, the **genetic change** of EICA can be implemented in adaptive dynamics |
| Bottom-up regulation (BUR) | Adaptation (A) | The **cross-guild interaction process** of BUR can promote invasion through A |
| Bottom-up regulation (BUR) | Empty niche (EN) | The **cross-guild interaction process** of BUR can promote invasion through EN |
| Bottom-up regulation (BUR) | Environmental heterogeneity (EH) | The **cross-guild interaction process** of EH is a special case of BUR |
| Bottom-up regulation (BUR) | Habitat filtering (HF) | The **cross-guild interaction process** of HF is a special case of BUR |
| Bottom-up regulation (BUR) | Increased resource availability (IRA) | The **cross-guild interaction process** of IRA is a special case of BUR |
| Bottom-up regulation (BUR) | New associations (NA) | The New association (NA) can potentially be with an abiotic resource, i.e. through a **cross-guild interaction process** |
| Bottom-up regulation (BUR) | Opportunity windows (OW) | The **cross-guild interaction process** of BUR can promote invasion through an OW |
| Colonization-competition trade-off / patch dynamics (CCT/PD) | Dynamic equilibrium (DE) | The random fluctuation leading to changes in **within-guild interaction process** can break the trade-off with **dispersal** in CCT/PT. |
| Colonization-competition trade-off / patch dynamics (CCT/PD) | Increased resource availability (IRA) | The increase in resource from IRA gives an advantage through the **cross-guild interaction process** that breaks the trade-off with **dispersal** in CCT/PT. |
| Colonization-competition trade-off / patch dynamics (CCT/PD) | Opportunity windows (OW) | OW can give a temporary advantage through the **cross-guild interaction process** that can break the trade-off with **dispersal** in CCT/PT. |
| Competitive exclusion principle (CE) | Darwin's naturalization (DN) | These models assume the same effect of **within-guild biotic interactions**. |
| Competitive exclusion principle (CE) | Limiting similarity (LS) | These models assume the same effect of **within-guild biotic interactions**. |

| | | |
|---|---|---|
| Enemy-mediated coexistence (EMC) | Enemy inversion (EI) | The same **cross-guild biotic interactions** explain both models. |
| Enemy-mediated coexistence (EMC) | Enemy of my enemy (EE) | The same **cross -guild biotic interactions** explain both models. |
| Enemy-mediated coexistence (EMC) | Enemy reduction (ERD) | The same **cross -guild biotic interactions** explain both models. |
| Enemy-mediated coexistence (EMC) | Enemy release (ER) | The same **cross -guild biotic interactions** explain both models. |
| Enemy-mediated coexistence (EMC) | Resource-enemy release (RER) | The same **cross -guild biotic interactions** occur in both models, although RER also assumes that **abiotic interaction** modulates the effect this process. |
| Equalizing/stabilizing criteria (ESC) | Empty niche (EN) | The presence of empty niches from **interaction processes** is a stabilizing mechanism. |
| Equalizing/stabilizing criteria (ESC) | Evolution of increased competitive ability (EICA) | EICA breaks the equalizing mechanisms resulting from **intra-guild interactions** from ESC |
| Equalizing/stabilizing criteria (ESC) | Limiting similarity (LS) | LS implies that stabilizing mechanisms are required for coexistence in **intra-guild interactions**. |
| Facilitation-based theory (FBT) | Missed mutualisms (MM) | The **cross-guild biotic interactions** of MM are described by FBT. |
| Genetic feedback (GF) | Evolution of increased competitive ability (EICA) | The same **genetic changes** explain both models. |
| Intermediate disturbance hypothesis (IDH) | Empty niche (EN) | The **drift** occurring in IDH can favor biological invasions through the **within-guild biotic interactions** described by EN. |
| Janzen-Connell effects (JC) | Enemy inversion (EI) | The same **cross-guild biotic interactions** explain both models. |
| Janzen-Connell effects (JC) | Enemy of my enemy (EE) | The same **cross-guild biotic interactions** explain both models. |
| Janzen-Connell effects (JC) | Enemy reduction (ERD) | The same **cross-guild biotic interactions** explain both models. |
| Janzen-Connell effects (JC) | Enemy release (ER) | The same **cross-guild biotic interactions** explain both models. |
| Neutral theory (NeT) | Propagule pressure (PP) | The **propagule pressure** of PP is a major process of NeT. |
| Niche theory (NiT) | Adaptation (A) | A is based on **interaction processes**, therefore falls under the NiT umbrella. |
| Niche theory (NiT) | Biotic Indirect Effect (BID) | BID is based on **interaction processes**, therefore falls under the NiT umbrella. |

| | | |
|---|---|---|
| Niche theory (NiT) | Dynamic equilibrium (DE) | DE is based on competition, i.e. **within-guild interaction process**, therefore falls under the NiT umbrella. |
| Niche theory (NiT) | Empty niche (EN) | EN is based on **interaction processes**, therefore falls under the NiT umbrella. |
| Niche theory (NiT) | Environmental heterogeneity (EH) | EH is based on **abiotic interactions**, therefore falls under the NiT umbrella. |
| Niche theory (NiT) | Habitat filtering (HF) | HF is based on **abiotic interactions**, therefore falls under the NiT umbrella of **interaction processes**. |
| Niche theory (NiT) | Increased resource availability (IRA) | IRA is based on **interaction processes**, therefore falls under the NiT umbrella. |
| Niche theory (NiT) | New associations (NA) | NA is based on **within-guild interactions**, therefore falls under the NiT umbrella of **interaction processes**. |
| Niche theory (NiT) | Novel weapon (NW) | NW is based on **within-guild interactions**, therefore falls under the NiT umbrella of **interaction processes**. |
| Niche theory (NiT) | Opportunity windows (OW) | OW considers that the **interaction processes**, therefore of NiT are expressed for a limited time in a certain way that favors biological invasions. |
| Niche theory (NiT) | Specialist-generalist (SG) | SG is based on **cross-guild interactions**, therefore falls under the NiT umbrella. |
| R* theory (R*) | Adaptation (A) | An alien species pre-adapted (A) to an exotic environment will have a low R*, and therefore benefit from **abiotic interactions**. |
| R* theory (R*) | Empty niche (EN) | The absence of native species with low R* allow an alien species to establish through **abiotic interactions**, which corresponds to EN. |
| R* theory (R*) | Resource-enemy release (RER) | RER assumes that release from an enemy will lower the R* of an alien species, i.e. modify **abiotic interactions**. |
| Relative nonlinearity of competition (RNC) | Increased resource availability (IRA) | Resource fluctuation under RNC can create IRA and modify **abiotic interactions**. |
| Relative nonlinearity of competition (RNC) | Opportunity windows (OW) | Resource fluctuation under RNC can create OW by modifying **abiotic interactions**. |
| Spatial storage effect (SSE) | Environmental heterogeneity (EH) | Spatial variability in **abiotic interactions** process explains both models. |
| Spatial storage effect (SSE) | Increased resource availability (IRA) | SSE can enable an alien species to wait for IRA, i.e. to benefit from favorable **abiotic interactions**. |

| | | |
|---|---|---|
| Spatial storage effect (SSE) | Opportunity windows (OW) | SSE can enable an alien species to wait for OW, i.e. to benefit from favorable **interaction processes**. |
| Species pool hypothesis (SPH) | Global competition (GC) / Sampling (SP) | The **colonization pressure** involved in GC/SP is determined by a species pool of alien species, as described by SPH. |
| Species sorting (SS) | Adaptation (A) | A can be based on **abiotic interactions**, and can therefore occur under SS. |
| Species sorting (SS) | Empty niche (EN) | EN can be based on **abiotic interactions**, and can therefore occur under SS. |
| Species sorting (SS) | Environmental heterogeneity (EH) | EH is based on **abiotic interactions**, and can therefore occur under SS. |
| Species sorting (SS) | Habitat filtering (HF) | HF is based on **abiotic interactions**, and can therefore occur under SS. |
| Species sorting (SS) | Increased resource availability (IRA) | IRA can be based on **abiotic interactions**, and can therefore occur under SS. |
| Species sorting (SS) | Opportunity windows (OW) | OW can be based on **abiotic interactions**, and can therefore occur under SS. |
| Species-energy theory (SET) | Propagule pressure (PP) | PP can affect immigration rates under SET through **propagule pressure**. |
| Succession theory (ST) | Dynamic equilibrium (DE) | ST can represent the fluctuations changing the **within-guild interactions** of DE. |
| Succession theory (ST) | Empty niche (EN) | Different stages of ST can give different weights to the **interaction processes** encompassed by EN. |
| Succession theory (ST) | Increased resource availability (IRA) | ST can modify **abiotic interactions** and create IRA. |
| Succession theory (ST) | Opportunity windows (OW) | ST can modify the different **interaction processes** and create OW. |
| Temporal storage effect (TS) | Environmental heterogeneity (EH) | Asynchronous temporal fluctuations can generate environmental heterogeneity and modify **abiotic interactions**. |
| Temporal storage effect (TS) | Increased resource availability (IRA) | TS can enable an alien species to wait for IRA, i.e. favorable **abiotic interactions**. |
| Temporal storage effect (TS) | Opportunity windows (OW) | TS can enable an alien species to wait for OW, i.e. favorable **interaction processes**. |
| Theory of island biogeography (TIB) | Propagule pressure (PP) | The **propagule pressure** process of PP can affect the immigration rates considered by TIB. |
| Top-down regulation (TDR) | Enemy inversion (EI) | The same **cross-guild biotic interactions** explain both models. |
| Top-down regulation (TDR) | Enemy of my enemy (EE) | The same **cross-guild biotic interactions** explain both models. |

| | | |
|---|---|---|
| Top-down regulation (TDR) | Enemy reduction (ERD) | The same **cross-guild biotic interactions** explain both models. |
| Top-down regulation (TDR) | Enemy release (ER) | The same **cross-guild biotic interactions** explain both models. |
| Top-down regulation (TDR) | New associations (NA) | The **cross-guild biotic interactions** between an alien species and native prey or predators assumed by NA can be described by TDR. |
| Top-down regulation (TDR) | Resource-enemy release (RER) | The **cross-guild biotic interactions** of TDR are present modulated by **abiotic interactions** in RER. |
| Top-down regulation (TDR) | Specialist-generalist (SG) | The **cross-guild biotic interactions** considered by SG are a special case of TDR. |

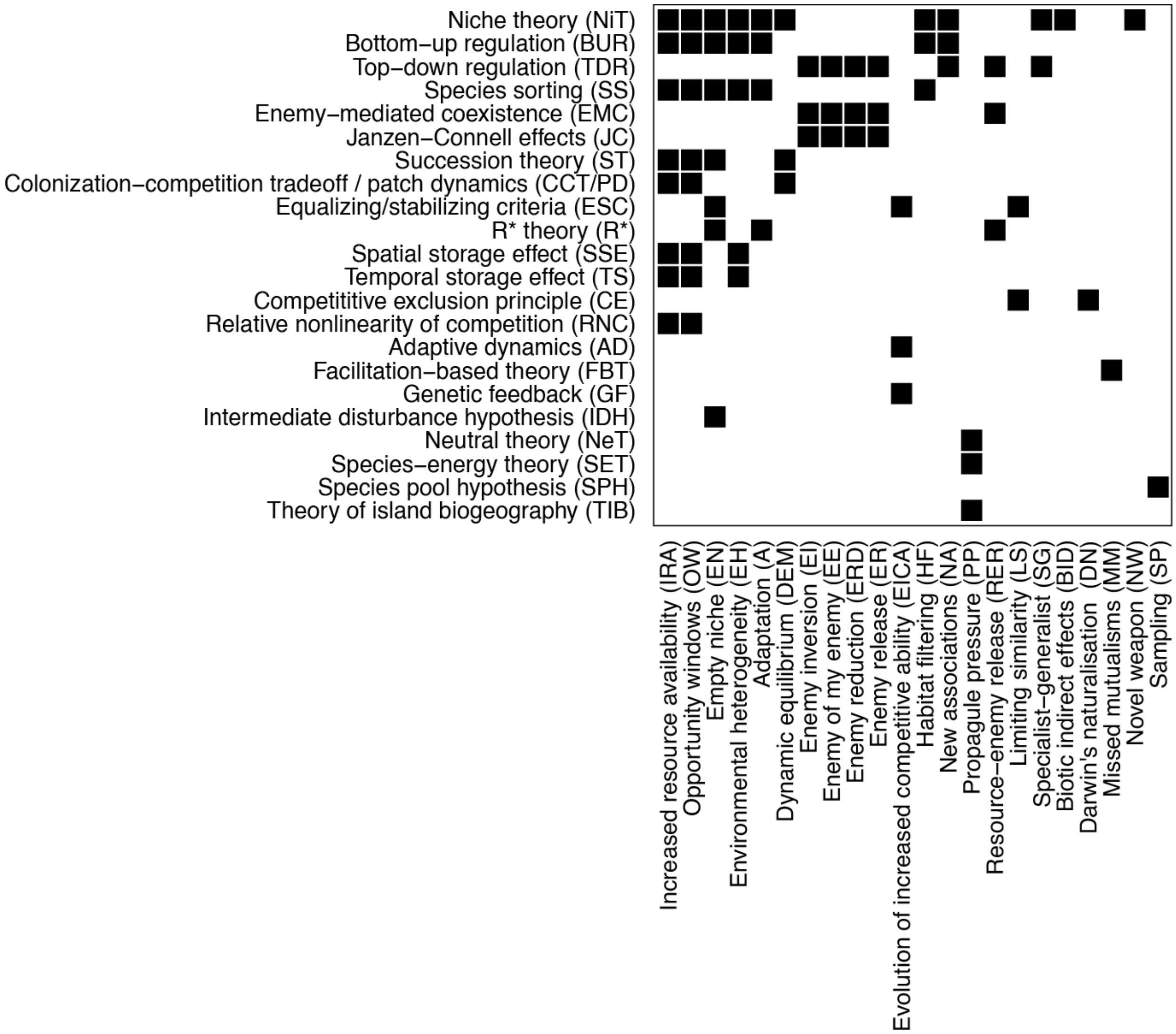


**Figure S1.** Bipartite network represented to show nestedness.

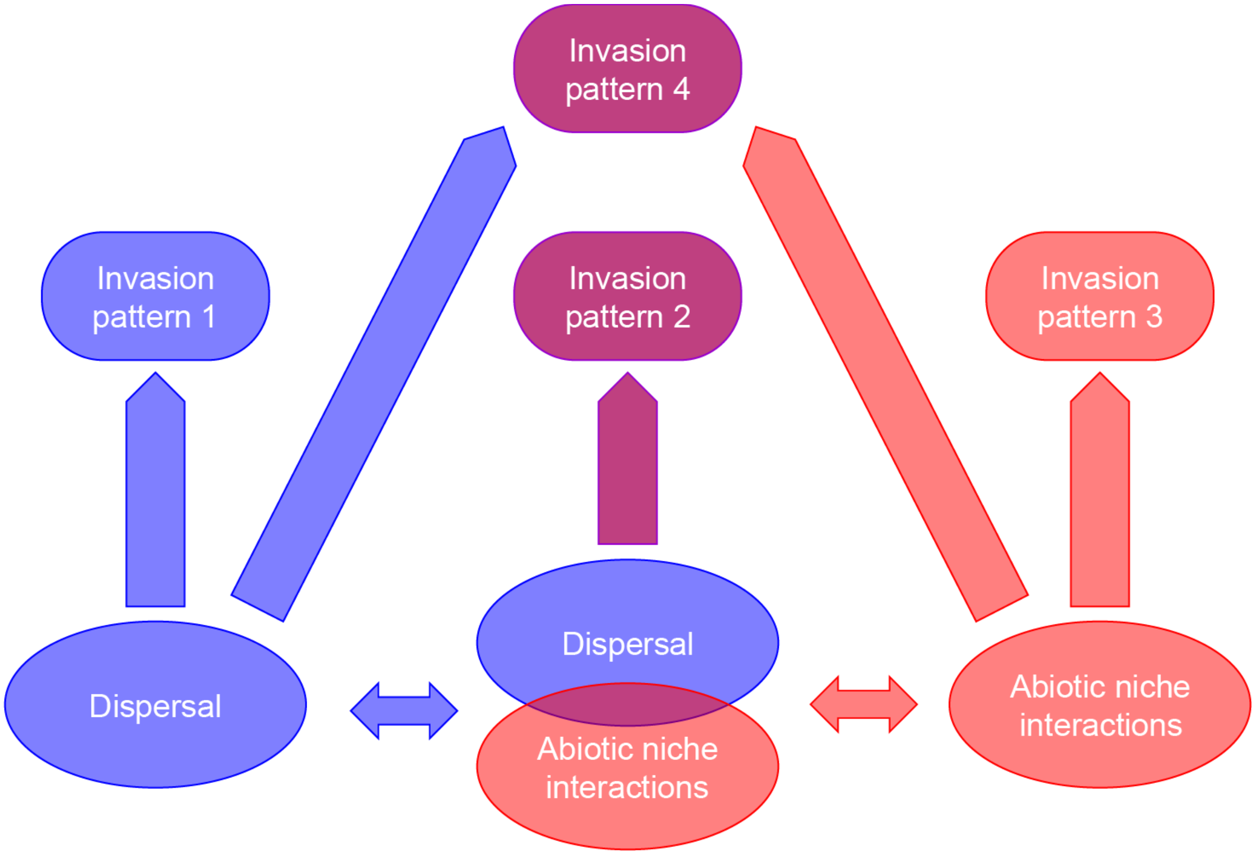


**Figure S2.** Hypothetical example of the relationship between process-based invasion models and invasion patterns. The dispersal and the abiotic niche interaction processes are related to the combination of dispersal and abiotic niche interactions, but not to each other, since they are independent processes. If we consider four invasion patterns (such as human commensalism, island susceptibility, etc.) that are generated by these three process combinations, invasion patterns 3 and 4 are related to each other because they are both generated by abiotic niche interactions, and so are patterns 1 and 4, generated by dispersal. They are partially related to the invasion pattern 2 since it is generated by the combination of dispersal and abiotic niche interactions. The invasion patterns 1 and 3 are not related to each other.

## References

Alpert, P. 2006. The advantages and disadvantages of being introduced. Biological Invasions 8:1523–1534.

Armstrong, R. A., and R. McGehee. 1980. Competitive exclusion. The American Naturalist 115:151–170.

Blossey, B., and R. Notzold. 1995. Evolution of increased competitive ability in invasive

nonindigenous plants: a hypothesis. Journal of Ecology 83:887–889.

Blumenthal, D. M. 2006. Interactions between resource availability and enemy release in plant invasion. Ecology Letters 9:887–895.

Bruno, J. F., J. J. Stachowicz, and M. D. Bertness. 2003. Inclusion of facilitation into ecological theory. Trends in Ecology & Evolution 18:119–125.

Callaway, R. M., and W. M. Ridenour. 2004. Novel weapons: invasive success and the evolution of increased competitive ability. Frontiers in Ecology and the Environment 2:436–443.

Callaway, R. M., G. C. Thelen, A. Rodriguez, and W. E. Holben. 2004. Soil biota and exotic plant invasion. Nature 427:731.

Chase, J. M., and M. A. Leibold. 2003. Ecological niches: linking classical and contemporary approaches. University of Chicago Press, Chicagol, IL.

Chesson, P. 2000a. Mechanisms of maintenance of species diversity. Annual review of Ecology and Systematics 31:343–366.

Chesson, P. 2000b. General theory of competitive coexistence in spatially-varying environments. Theoretical Population Biology 58:211–237.

Colautti, R. I., A. Ricciardi, I. A. Grigorovich, and H. J. MacIsaac. 2004. Is invasion success explained by the enemy release hypothesis? Ecology Letters 7:721–733.

Connell, J. H. 1970. On the role of natural enemies in preventing competitive exclusion in some marine animals and in rain forest trees. Pages 298–312 *in* P. J. Den Boer and G. R. Gradwell, editors. Dynamics of populations. Pudoc, Wageningen.

Connell, J. H. 1978. Diversity in tropical rain forests and coral reefs. Science 199:1302–1310.

Crawley, M. J., S. L. Brown, M. S. Heard, and G. R. Edwards. 1999. Invasion-resistance in experimental grassland communities: species richness or species identity? Ecology Letters 2:140–148.

Darwin, C. 1859. On the origin of species by means of natural selection. John Murray, London, UK.

Duncan, R. P., and P. A. Williams. 2002. Ecology: Darwin's naturalization hypothesis challenged. Nature 417:608–609.

Eppinga, M. B., M. Rietkerk, S. C. Dekker, P. C. De Ruiter, W. H. Van der Putten, and W. H. Van der Putten. 2006. Accumulation of local pathogens: a new hypothesis to explain exotic plant invasions. Oikos 114:168–176.

Fussmann, G. F., M. Loreau, and P. A. Abrams. 2007. Eco-evolutionary dynamics of communities and ecosystems. Functional Ecology 21:465–477.

Gause, G. F. 1934. The struggle for existence. The Williams & Wilkins company, Baltimore.

Gilpin, M. E. 1975. Limit cycles in competition communities. The American Naturalist 109:51–60.

Gravel, D., C. D. Canham, M. Beaudet, and C. Messier. 2006. Reconciling niche and neutrality: the continuum hypothesis. Ecology Letters 9:399–409.

Grime, J. P. 1973. Competitive exclusion in herbaceous vegetation. Nature 242:344–347.

Holt, R. D., J. Grover, and D. Tilman. 1994. Simple rules for interspecific dominance in systems with exploitative and apparent competition. The American Naturalist 144:741–771.

Holyoak, M., M. A. Leibold, and R. D. Holt, editors. 2005. Metacommunities: spatial dynamics and ecological communities. University of Chicago Press, Chicagol, IL.

Hubbell, S. P. 2001. The unified neutral theory of biodiversity and biogeography (MPB-32)(monographs in population biology). Princeton University Press, Princeton, NJ.

Huston, M. 1979. A general hypothesis of species diversity. The American Naturalist 113:81–101.

Janzen, D. H. 1970. Herbivores and the number of tree species in tropical forests. The American Naturalist 104:501–528.

Johnstone, I. M. 1986. Plant invasion windows: a time-based classification of invasion potential. Biological Reviews 61:369–394.

Jones, C. G., J. H. Lawton, and M. Shachak. 1994. Organisms as ecosystem engineers. Pages 130–147 Ecosystem Management. Springer, New York, NY.

Keane, R. M., and M. J. Crawley. 2002. Exotic plant invasions and the enemy release hypothesis. Trends in Ecology & Evolution 17:164–170.

Latombe, G., C. Hui, and M. A. McGeoch. 2015. Beyond the continuum: a multi-dimensional phase space for neutral–niche community assembly. Proceedings of the Royal Society B: Biological Sciences 282:20152417.

Leibold, M. A., and J. M. Chase, editors. 2017. Metacommunity ecology. Princeton University Press, Princeton, NJ.

Levins, R., and D. Culver. 1971. Regional coexistence of species and competition between rare species. Proceedings of the National Academy of Sciences 68:1246–1248.

Lockwood, J. L., P. Cassey, and T. Blackburn. 2005. The role of propagule pressure in explaining species invasions. Trends in Ecology & Evolution 20:223–228.

MacArthur, R. 1970. Species packing and competitive equilibrium for many species. Theoretical Population Biology 1:1–11.

MacArthur, R. H., and E. O. Wilson. 1967. The Theory of Island Biogeography. Princeton University Press, Princeton, NJ.

MacArthur, R., and R. Levins. 1967. The limiting similarity, convergence, and divergence of coexisting species. The American Naturalist 101:377–385.

Matson, P. A., and M. D. Hunter. 1992. Special feature: The relative contributions to top-down and bottom-up forces in population and community ecology. Ecology 73:723.

Melbourne, B. A., H. V Cornell, K. F. Davies, C. J. Dugaw, S. Elmendorf, A. L. Freestone, R. J. Hall, S. Harrison, A. Hastings, and M. Holland. 2007. Invasion in a heterogeneous world: resistance, coexistence or hostile takeover? Ecology Letters 10:77–94.

Mitchell, C. E., A. A. Agrawal, J. D. Bever, G. S. Gilbert, R. A. Hufbauer, J. N. Klironomos, J. L. Maron, W. F. Morris, I. M. Parker, and A. G. Power. 2006. Biotic interactions and plant invasions. Ecology Letters 9:726–740.

Oksanen, L., S. D. Fretwell, J. Arruda, and P. Niemela. 1981. Exploitation ecosystems in gradients of primary productivity. The American Naturalist 118:240–261.

Pickett, S. T. A., S. L. Collins, and J. J. Armesto. 1987. Models, mechanisms and pathways of succession. The Botanical Review 53:335–371.

Pimentel, D. 1968. Population regulation and genetic feedback. Science 159:1432–1437.

Sher, A. A., and L. A. Hyatt. 1999. The disturbed resource-flux invasion matrix: a new framework for patterns of plant invasion. Biological Invasions 1:107–114.

Taylor, D. R., L. W. Aarssen, and C. Loehle. 1990. On the relationship between r/K selection and environmental carrying capacity: a new habitat templet for plant life history strategies. Oikos 58:239–250.

Tilman, D. 1982. Resource competition and community structure. Princeton university press, Princeton, NJ.

Tilman, D. 2004. Niche tradeoffs, neutrality, and community structure: a stochastic theory of resource competition, invasion, and community assembly. Proceedings of the National academy of Sciences 101:10854–10861.

Wright, D. H. 1983. Species-energy theory: an extension of species-area theory. Oikos 41:496–506.
