# Supplementary material for: Mechanistic reconciliation of community and invasion ecology
Source: Ecosphere. 2021 Feb 10;12(2):e03359. doi: 10.1002/ecs2.3359 (PMC8647914; doi:10.1002/ecs2.3359)
Supplement: Supplementary file 3 — Appendix S3 [file ECS2-12-e03359-s003.pdf]

Supporting Information.

Latombe G, Richardson DM, McGeoch MA, Altwegg R, Catford JA, Chase JM, Courchamp F, Esler KJ, Jeschke JM, Landi P, Measey J, Midgley GF, Minoarivelo HO, Rodger JA, Hui C. Mechanistic reconciliation of community and invasion ecology. *Ecosphere.*

Appendix S3. Supplementary tables and figures for the flexible characterization

Table S1. Justification of process inclusion for the community model considered as process-based under the flexible characterization only.

ID	Community model	Reference(s)	Dispersal / Propagule pressure / Colonization pressure	Drift	Interactions			Genetic changes (speciation, adaptation)
					Abiotic interactions	Biotic interactions		
						Within-guild	Cross-Guild	
C21	Priority effect (PE)	(Fukami 2010, 2015)				Priority effects can be caused by competition	Priority effects can be caused by predator-prey interactions	

Table S2. Justification of process inclusion of each of the invasion models considered as process-based under the flexible characterization only

ID	Invasion model	Reference(s)	Dispersal / Propagule pressure / Colonization pressure	Drift	Interactions			Genetic changes (speciation, adaptation)
					Abiotic interactions	Biotic interactions		
						Within-guild	Cross-Guild	
I2	Biotic acceptance aka "the rich get richer" (BA)	(Stohlgren et al. 1998)			Environment can be species-rich due to diverse abiotic conditions, offering potential niches to alien species		Species-rich environments are more likely to contain species that can be resources for alien species	
I4	Biotic resistance aka diversity-invasibility hypothesis (BR)	(Elton 1958, Levine and D'Antonio 1999)				Species-rich environments are more likely to contain competitors	Species-rich environments are more likely to contain predators	
I6	Disturbance (DS)	(Elton 1958, Hobbs and Huenneke 1992)		Disturbance represents drift				

I21	Invasional meltdown (IM)	(Simberloff and Von Holle 1999, Sax et al. 2007)			Alien species can change abiotic conditions, e.g. the frequency of fires	The presence of mutualist alien species can facilitate further invasions	Alien species can be resources for other alien species	
I22	Island susceptibility hypothesis (ISH)	(Jeschke 2008, Moser et al. 2018)				Islands have fewer species and therefore fewer competitors	Islands have fewer species and therefore fewer predators	

Table S3. Associations between process-based community and invasion models, using the flexible characterization. The processes linking community and invasion models are indicated in bold. Shading is only used to distinguish between community models.

Community models/theories	Invasion models	Justification
Bottom-up regulation (BUR)	Biotic acceptance aka "the rich get richer" (BA)	BUR involves the **cross-guild interaction processes** leading to the resource consumption potentially involved in BA
Bottom-up regulation (BUR)	Invasional meltdown (IM)	Alien species can be resources for other alien species, i.e. **cross-guild interactions**.
Competitive exclusion principle (CE)	Biotic resistance aka diversity-invasibility hypothesis (BR)	BR can be based on the **within-guild biotic interactions** of CE.
Competitive exclusion principle (CE)	Island susceptibility hypothesis (ISH)	If richness is lower in islands, the absence of **within-guild biotic interactions** as described by CE can lead to ISH.
Ecosystem engineering (EE)	Invasional meltdown (IM)	The effect of ecosystem engineers on the **abiotic** environment can lead to IM.
Enemy-mediated coexistence (EMC)	Biotic acceptance aka "the rich get richer" (BA)	The **cross-guild interactions** of EMC can lead to BA.
Enemy-mediated coexistence (EMC)	Biotic resistance aka diversity-invasibility hypothesis (BR)	The **cross-guild interaction**s of EMC can lead to BR.
Equalizing/stabilizing criteria (ESC)	Biotic resistance aka diversity-invasibility hypothesis (BR)	ESC can explain BR as the presence of many species decreases the chance for equalizing mechanisms through **within-guild interactions**.
Equalizing/stabilizing criteria (ESC)	Island susceptibility hypothesis (ISH)	Lower richness in islands leads to increased stabilizing mechanisms through **within-guild interactions**, and therefore ISH.
Facilitation-based theory (FBT)	Biotic acceptance aka "the rich get richer" (BA)	High species richness leads to more change for facilitation mechanisms, i.e. **biotic interactions**, and therefore BA.
Facilitation-based theory (FBT)	Invasional meltdown (IM)	IM can be caused by facilitation processes occurring between alien species, i.e. **biotic interactions**.

Intermediate disturbance hypothesis (IDH)	Disturbance (DS)	Both IDH and DS are based on **drift processes**.
Janzen-Connell effects (JC)	Biotic resistance aka diversity-invasibility hypothesis (BR)	High species richness can promote the **biotic interactions** of JC, therefore preventing invasions.
Neutral theory (NeT)	Disturbance (DS)	Disturbance (**drift**) processes in NeT lead to high fluctuations species relative abundance, providing opportunities for invasions.
Niche theory (NiT)	Biotic acceptance aka “the rich get richer” (BA)	BA is based on **interaction processes**.
Niche theory (NiT)	Biotic resistance aka diversity-invasibility hypothesis (BR)	BR is based on **interaction processes**.
Niche theory (NiT)	Invasional meltdown (IM)	IM occur through **interaction processes**.
Niche theory (NiT)	Island susceptibility hypothesis (ISH)	ISH can be due to specific **interaction processes** in the presence of specific biota.
Priority effect (PE)	Invasional meltdown (IM)	IM can occur because of the **interaction processes** of PE.
Priority effect (PE)	Opportunity windows (OW; fluctuating resources)	The **interaction processes** of PE can provide OW.
R* theory (R*)	Biotic resistance aka diversity-invasibility hypothesis (BR)	High species richness increase the chances of having species with low R*, leading to BR due to **within-guild interactions**.
R* theory (R*)	Island susceptibility hypothesis (ISH)	Low richness on islands decrease the chances of having species with low R*, leading to ISH due to **within-guild interactions**.
Species pool hypothesis (SPH)	Island susceptibility hypothesis (ISH)	All the processes involved in SPH can lead to ISH, such as specific regional **interaction processes** resulting from a specific history of **genetic changes**.
Species sorting (SS)	Biotic acceptance aka “the rich get richer” (BA)	SS and BA are defined by **abiotic interaction processes**.
Stochastic niche theory (SN)	Disturbance (DS)	**Drift** in SN can explain DS.

Succession theory (ST)	Invasional meltdown (IM)	The **interaction processes** involved in ST can result in IM.
Theory of island biogeography (TIB)	Island susceptibility hypothesis (ISH)	Differences in **propagule pressure** can explain ISH under the TIB.
Top-down regulation (TDR)	Biotic resistance aka diversity-invasibility hypothesis (BR)	High richness increases the probability of presence if predators, leading to BR through **cross-guild interactions**.

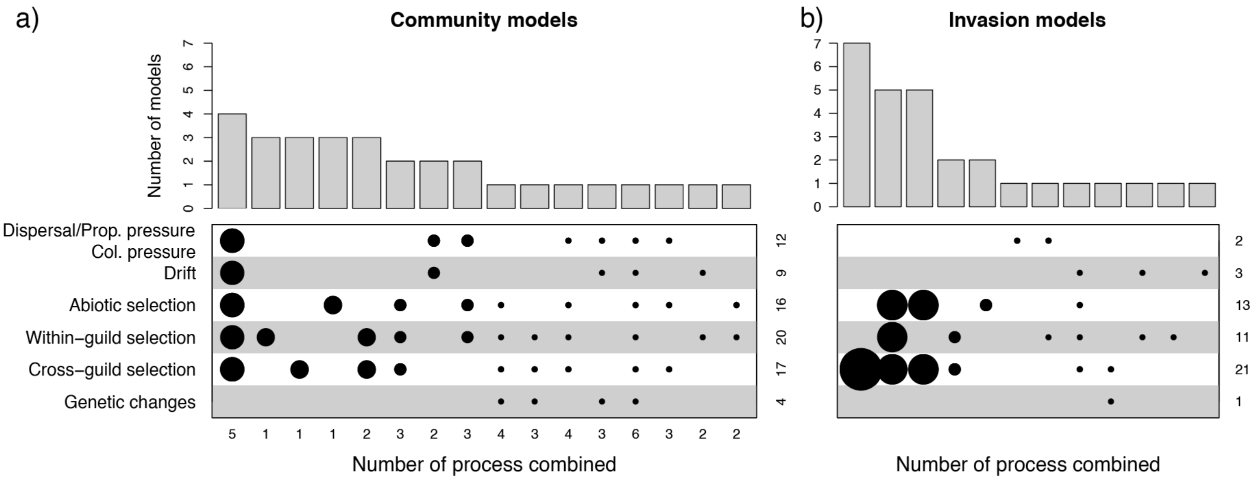

Figure S1. Summary of combinations of processes characterizing (a) the community models (n = 30) and (b) the invasion models (n = 28) using the flexible characterization. The lower plots indicate the combinations of processes that were identified, ordered by the number of models characterized by a specific combination (processes combined in a model are represented by the circles in a single column, and the number of combined processes is also indicated by the numbers at the bottom of the plots). The numbers on the right of the graphs represent the number of models that include each process. The size of the circles and the upper bar plot both indicate this number (the bar plot was used to better visualize the skewness of the distributions).

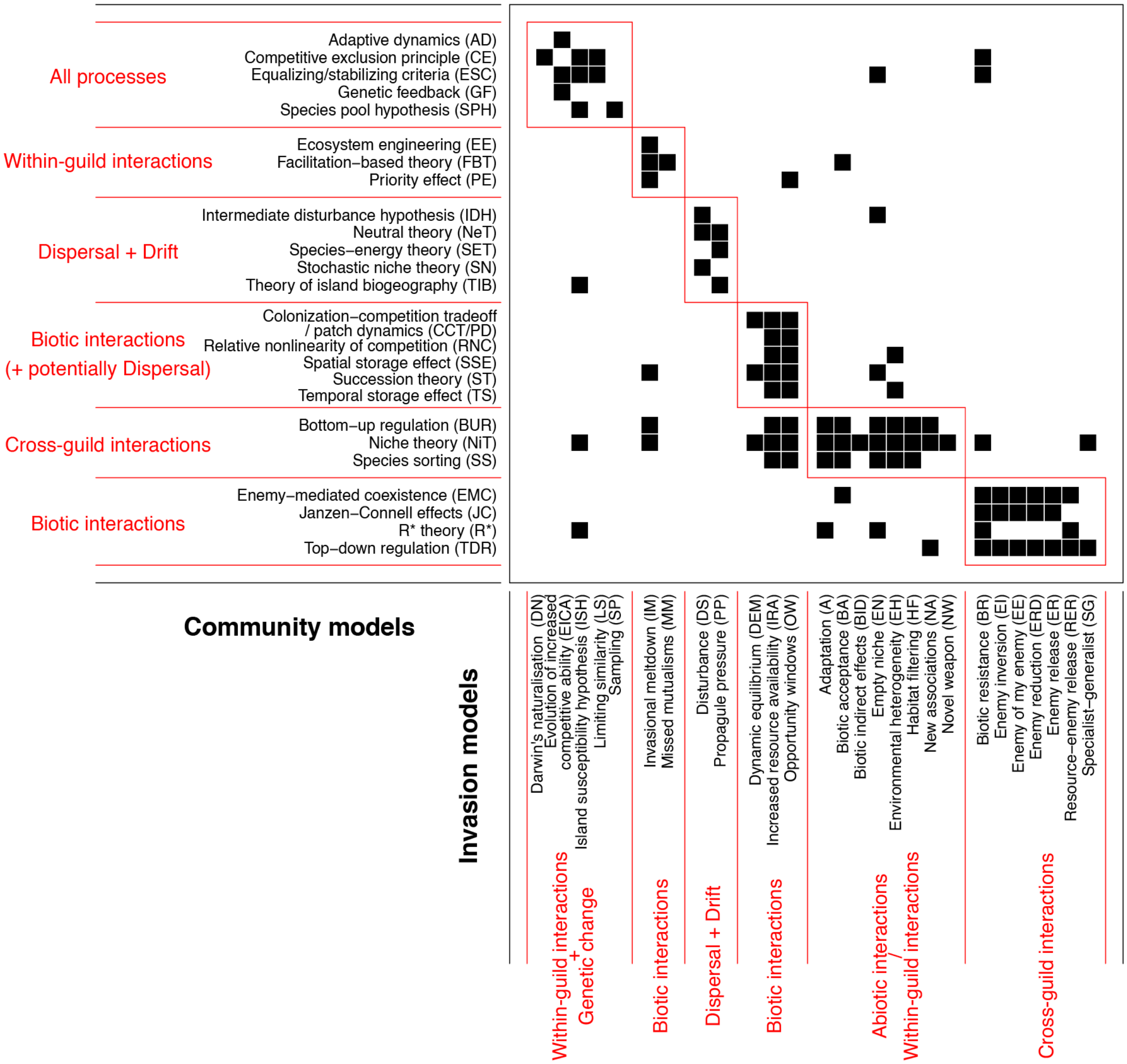

Figure S2. Relationship between community (rows) and invasion (columns) models presented as a bipartite network resulting from the flexible characterization. The modules were identified using the Dormann-Strauss algorithm. The main processes characterizing each module are indicated in red: "/" indicates that at least one process characterizes the models, whereas "+" indicates that the processes are combined in the models.

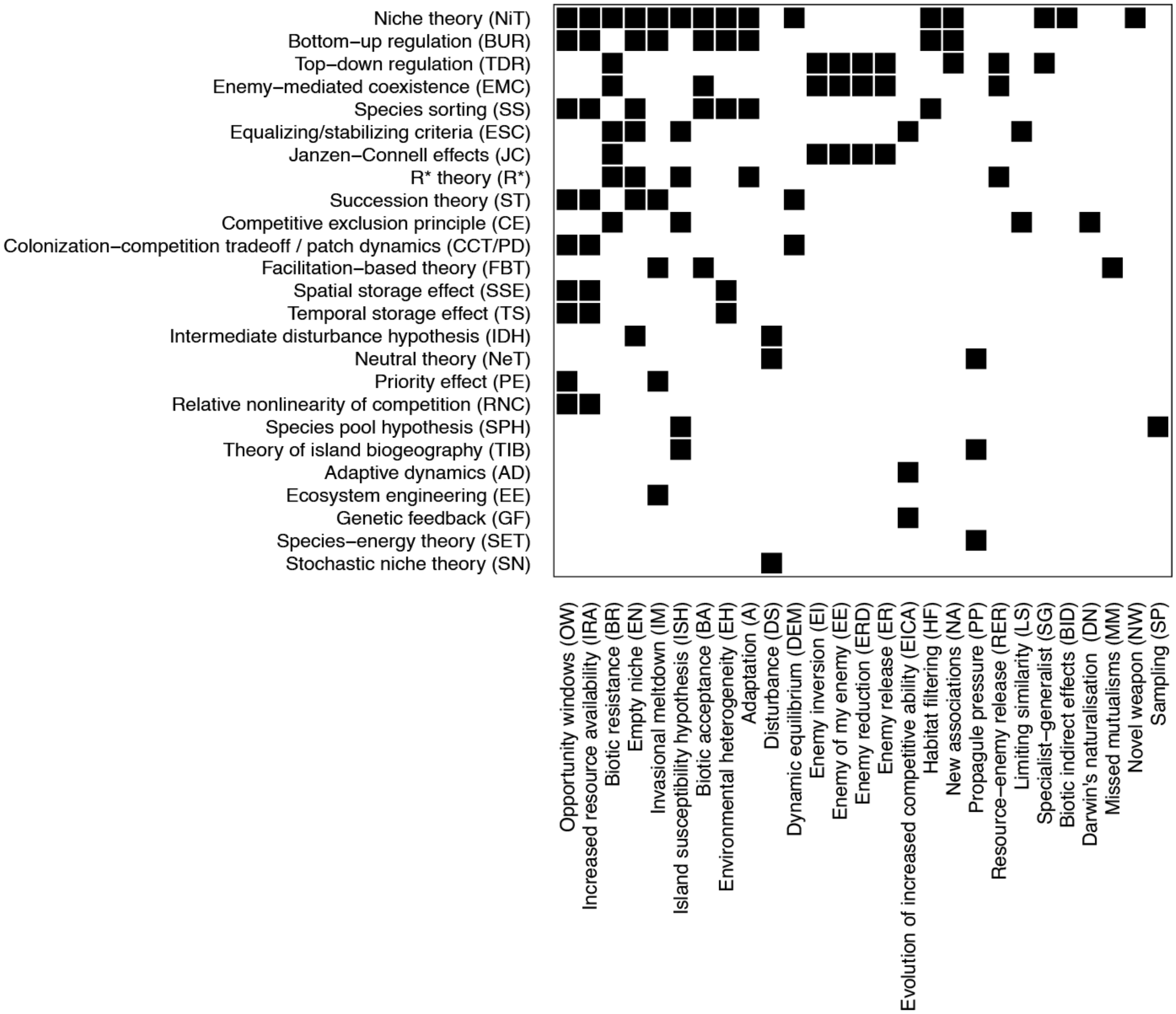

Figure S3. Bipartite network resulting from the flexible characterization represented to show nestedness.

References

Elton, C. S. 1958. The ecology of invasions by plants and animals. Chapman & Hal, London, UK.

Fukami, T. 2010. Community assembly dynamics in space. Pages 45–54 *in* H. Verhoef and P. Morin, editors. Community ecology: processes, models, and applications. Oxford University Press, Oxford, UK.

Fukami, T. 2015. Historical contingency in community assembly: integrating niches, species

pools, and priority effects. Annual Review of Ecology, Evolution, and Systematics 46:1–23.

Hobbs, R. J., and L. F. Huenneke. 1992. Disturbance, diversity, and invasion: implications for conservation. Conservation Biology 6:324–337.

Jeschke, J. M. 2008. Across islands and continents, mammals are more successful invaders than birds. Diversity and Distributions 14:913–916.

Levine, J. M., and C. M. D'Antonio. 1999. Elton revisited: a review of evidence linking diversity and invasibility. Oikos 81:15–26.

Moser, D., B. Lenzner, P. Weigelt, W. Dawson, H. Kreft, J. Pergl, P. Pyšek, M. van Kleunen, M. Winter, C. Capinha, P. Cassey, S. Dullinger, E. P. Economo, P. García-Díaz, B. Guénard, F. Hofhansl, T. Mang, H. Seebens, and F. Essl. 2018. Remoteness promotes biological invasions on islands worldwide. Proceedings of the National Academy of Sciences 115:9270–9275.

Sax, D. F., J. J. Stachowicz, J. H. Brown, J. F. Bruno, M. N. Dawson, S. D. Gaines, R. K. Grosberg, A. Hastings, R. D. Holt, and M. M. Mayfield. 2007. Ecological and evolutionary insights from species invasions. Trends in Ecology & Evolution 22:465–471.

Simberloff, D., and B. Von Holle. 1999. Positive interactions of nonindigenous species: invasional meltdown? Biological Invasions 1:21–32.

Stohlgren, T. J., K. A. Bull, Y. Otsuki, C. A. Villa, and M. Lee. 1998. Riparian zones as havens for exotic plant species in the central grasslands. Plant Ecology 138:113–125.
